# Millikelvin Intracellular Nanothermometry with Nanodiamonds

**DOI:** 10.1002/advs.202511670

**Published:** 2025-09-29

**Authors:** Maabur Sow, Jacky Mohnani, Genko Genov, Raphael Klevesath, Elisabeth Mayerhoefer, Yuliya Mindarava, Rémi Blinder, Soumen Mandal, Fabien Clivaz, Raúl B. Gonzalez, Daniel Tews, Christian Laube, Wolfgang Knolle, Amelie Jerlitschka, Farid Mahfoud, Oleg Rezinkin, Mateja Prslja, Yingke Wu, Pamela Fischer‐Posovszky, Martin B. Plenio, Susana F. Huelga, Tanja Weil, Anke Krueger, Gavin W. Morley, Oliver A. Williams, Steffen Stenger, Fedor Jelezko

**Affiliations:** ^1^ Institute for Quantum Optics Ulm University D‐89081 Ulm Germany; ^2^ Institute for Medical Microbiology and Hygiene Ulm University 89081 Ulm Germany; ^3^ Institute of Organic Chemistry University of Stuttgart 70569 Stuttgart Germany; ^4^ School of Physics and Astronomy Cardiff University Cardiff CF24 3AA UK; ^5^ Institute for Theoretical Physics Ulm University D‐89069 Ulm Germany; ^6^ Department of Paediatrics and Adolescent Medicine Ulm University D‐89075 Ulm Germany; ^7^ Leibniz Institute of Surface Engineering (IOM) 04318 Leipzig Germany; ^8^ Max Planck Institute for Polymer Research 55128 Mainz Germany; ^9^ Center for Integrated Quantum Science and Technology University of Stuttgart 70569 Stuttgart Germany; ^10^ Department of Physics University of Warwick Coventry CV4 7AL UK; ^11^ Center for Integrated Quantum Science and Technology (IQST) Ulm University 89081 Ulm Germany

**Keywords:** diamond, nanothermometry, nitrogen‐vacancy, quantum sensing, thermodynamics of life

## Abstract

Nanothermometry within living cells is an important endeavor in physics, as the mechanisms of heat diffusion in such complex and dynamic environments remain poorly understood. In biology, nanothermometry may offer new insights into cellular biology and open new avenues for drug‐discovery. Previous studies using various nanothermometers have reported temperature variations of up to several Kelvins during metabolic stimulation, but these findings have remained controversial as they appear to contradict the law of heat diffusion in the presence of heating rates that are consistent with physiological parameters. Here, nanodiamond nanothermometry are reported inside macrophages by measuring the optically detected magnetic resonance spectra of nitrogen‐vacancy centers. The spectra are analyzed when cells are metabolically stimulated and after cell death. It is shown that, in the experimental setting, the apparent spin resonant spectral shifts can be misinterpreted as temperature changes but are actually caused by electrical field changes on the nanodiamond's surface. These artifacts are addressed with optimized nanodiamonds and a more robust sensing protocol to measure temperature inside cells with precision down to 100 mK (52 mK outside cells). No significant temperature changes upon metabolic stimulation are found, a finding consistent with the implementation of the heat diffusion law and expected physiological heating rates.

## Graphical abstract

Nanothermometry in living cells is crucial for understanding heat diffusion and advancing cellular biology and drug discovery. Previous studies have reported controversial intracellular temperature variations. Using nanodiamond nanothermometry in macrophages, it is found that, in such experimental setting, apparent temperature changes are actually due to electrical field changes. Optimized methods achieve precise measurements, showing no significant temperature changes upon metabolic stimulation.

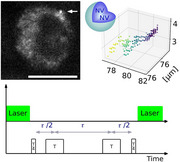

## 1 Introduction

Temperature in biology is paramount as biological processes depend on temperature, and yet little is known about temperature at the nanoscale. The magnitude and role of intracellular temperature gradients is still unclear as it is not only difficult to design robust nanothermometers, but also to model heat diffusion inside the cell. Still, nanoscale temperature sensing is an important field of research as intracellular gradients could play a role in cellular signaling; further, nanothermometry would improve our understanding of heat‐generating organelles such as mitochondria. In physics, nanothermometers could also provide valuable experimental data to invent and test new approaches to model heat generation and diffusion at the nanoscale.^[^ ^1^, ^2^ ^]^

In the past decades, different teams, using complementary approaches (fluorescence, Raman or thermocouple based) reported the existence of intracellular gradients (1–10 K differences between different cellular localizations or organelles metabolic states) and a 0.2–2 K temperature increase of individual cells upon metabolic stimulation.^[^ ^3^, ^4^, ^5^, ^6^, ^7^ ^]^ However, when considering the heat diffusion law and the most favorable assumptions, only temperature variations on the order of 10^−4^ K are expected.^[^ ^8^ ^]^

Great progress has been made on the theoretical side to take into account other sources of heat such as protonation/hydration processes in the mitochondria.^[^ ^9^ ^]^ There have been also significant experimental improvements to remove potential artifacts and measure thermal conductivity inside cells to apply more accurately the heat diffusion equation.^[^ ^10^, ^11^ ^]^ Nonetheless, no studies have been able to fully reconcile theory with experimental findings partially because a better understanding of nanothermometers is needed to design more robust and sensitive nanosensors.

**Figure 1:**
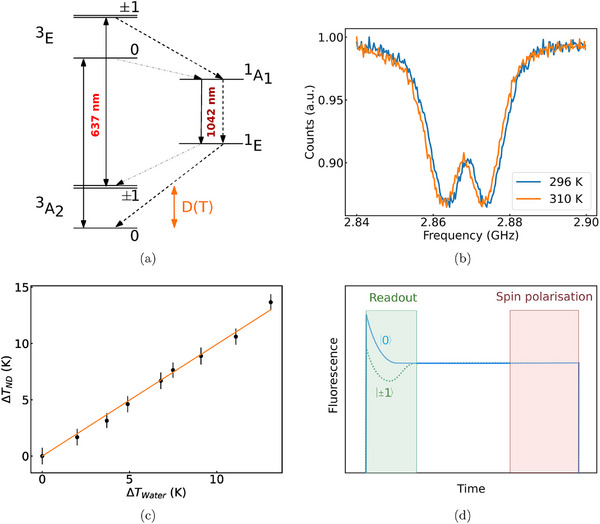
Principles of NV temperature sensing. a) Schematic of *NV* ^−^ fine electronic structure with the fine structure of the *NV* ^−^^3^A_2_ and ^3^E levels. The 637 nm ZPL corresponds to the transition from the ^3^E to the ^3^A_2_ triplet state. Inter‐system crossing can occur (from ^3^E to the ^1^A_1_ singlet state) thus leading to darker radiative transition (1042 nm). The excited triplet state ^3^E is not likely to undergo intersystem crossing to the ^1^A_1_ if the spin projection is |0〉 but for | ± 1〉, the intersystem crossing to a singlet state is more likely occur thus decreasing the NV fluorescence intensity (typically 5–20% for NVs in NDs).^[^ ^12^ ^]^ Following relaxation, the color center will convert back into the triplet ground state preferentially in the |0〉 state. This last step makes it possible to spin‐polarize the NV center to the |0〉 state just by several cycles of optical excitation. In our case, a 513 nm laser pulse of few microseconds was used.^[^ ^13^ ^]^ b) ODMR spectra of the same ND at two different temperatures (296 K‐23°C and 310 K‐37°C) illustrating the decrease of the ZFS as the temperature increases. c) ND temperature reading as the temperature of the water surrounding the ND is increased using an objective heater. The ZFS is extracted from the spectra using a double Lorentzian fit and taking the average of the center of the two dips (see example in Figure S.22, Supporting Information). The temperature of the water △*T*_*Water*_ measured with a thermocouple corresponds to ND readings △*T*_*ND*_ using the –74 kHz K^−1^ that was reported before as showed by the fit: *y* = *x*.^[^ ^14^ ^]^ Black dots: experimental data; orange line: fit. d) Schematic of a NV^−^ fluorescence pulse upon pulsed laser excitation. The readout window is typically 0.2–1 μ s depending on the laser power. Pulsed measurement can be performed on NV centers using laser pulses to read out the NV state. Then, following spin polarization, microwave pulses and interaction time is applied to detect a given physical quantity (e.g. magnetic field or temperature).^[^ ^15^ ^]^

Nanodiamonds (NDs) containing negative nitrogen‐vacancy (NV) centers have relatively low cytotoxicity and allow precise temperature readings in the mK range.^[^ ^16^, ^17^, ^18^ ^]^ The great sensitivity of NV‐NDs comes from the unique electronic structure of the fluorescent NV center and its remarkable photostability. Nanothermometry can be performed locally by exploiting its spin‐dependent fluorescence as the |*m*_*s*_ = ±1〉 is typically 30 % darker than the |*m*_*s*_ = 0〉 (from this point onward we denote |*m*_*s*_ = η〉 = |η〉).^[^ ^19^ ^]^ Thus, using continuous wave optically detected magnetic resonance (CW‐ODMR), one can measure the zero‐field splitting (ZFS, parameter D), which is linearly dependent on temperature (*dD*/*dT* = –74 kHz K^−1^) between 273.15 K and 325.15 K (**Figure** **1**).^[^ ^14^ ^]^

NV‐NDs have been used inside cells to measure temperature change induced by external heat sources such as infrared lasers with great precision (270 to 50 mK)^[^ ^17^, ^20^ ^]^ but when it comes down to measuring intracellular heat, errors remain significant with uncertainties from 0.24 to 1 K.^[^ ^6^, ^21^ ^]^

The precision can be improved by using pulsed measurements as they allow better control of the NVs by permitting not only noise correction (e.g. magnetic noise) but also avoiding optical and microwave power broadening.^[^ ^18^, ^22^ ^]^ Using such protocols, temperature precision down to 1 mK has been reported with a noise floor of 130 $mK/\sqrt{Hz}$ still, pulsed measurements for nanothermometry have not been used inside the cells for two main reasons. The first one relates to the fact that typical pulsed protocols require a well defined two‐level system for the entire duration of the measurement. To do so a static magnetic field (bias field) is used to split the | − 1〉 and | + 1〉 states but this is quite impractical with intracellular NDs as cells migrate and NDs may move or rotate inside the cell body.^[^ ^13^, ^18^, ^23^ ^]^ The second reason relates to the low brightness of single NVs, which makes them hard to distinguish from cell autofluorescence. Yet, single NVs offer the high ODMR contrast and great coherence properties that are essential for accurate pulsed measurements.

In this article, we report an important artifact affecting ND nanothermometry, which is manifested by fluctuations in the shape of the ODMR spectra and show that this can be used to investigate the variation of electrical fields on ND surfaces. We demonstrate that the impact of electric noise can be substantially reduced using dipeptide functionalization or low‐defect (nitrogen and vacancies) NDs. Then, we further improve the precision of our sensor with a pulse sequence designed to work with three‐level systems such as the NV spin, implemented with rectangular and robust rapid adiabatic passage (RAP) microwave pulses.^[^ ^26^ ^]^ Our approach is more robust against ND rotation/displacements, suitable for NDs containing multiple NVs and does not require the use of a bias field. We achieve 100 mK precision within a few minutes on immobilized NDs (1.1 $K/\sqrt{Hz}$) reaching 52 mK outside the cell and 100 mK inside the cells. Our intracellular nanothermometry measurements do not show any significant temperature change upon metabolic stimulation which supports the heat diffusion calculation previously reported.^[^ ^8^ ^]^

## 2 Results

### 2.1 Continuous Wave ODMR on Immobilized NDs

**Figure 2:**
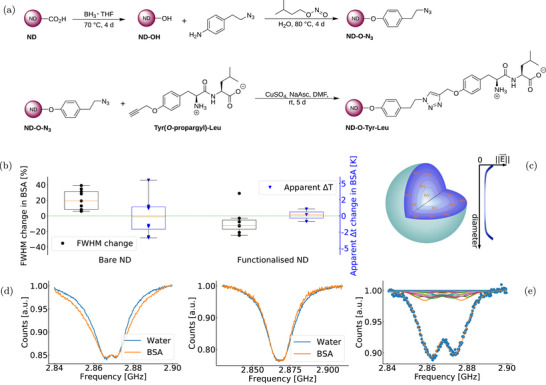
Effect of surface electrical fields on NDs' ODMR spectra. a) Functionalization of HPHT NDs. The carboxylic acid groups are reduced with borane‐tetrahydrofuran. Then, the hydroxyl groups are subsequently used to anchor a short aromatic linker molecule to the particle surface using diazonium coupling technology as reported in Merz et al.^[^ ^24^ ^]^ Finally, alkyne‐modified tyrosyl‐leucine dipeptide is covalently bound to azide functionalized particles ND‐O‐N_3_ by Cu(I)‐catalyzed “click” reaction giving dipeptide functionalized ND‐O‐Tyr‐Leu.^[^ ^25^ ^]^ b) Comparing the change of FWHM and apparent △*T* for the same set of NDs from water to BSA. Bare HPHT NDs (FWHM boxplot N = 9, △*T* boxplot N = 8) showed more variability than functionalized HPHT NDs (FWHM boxplot N = 7, △*T* boxplot N = 3). For all the ODMR spectra and more details, see Figure S.4 and S.9 (Supporting Information) and the Experiment Section. Each point represents a ND measurement, the box is from the first to the third quartile with the orange line being the median. The whisker lengths are 1.5 times the inter‐quartile range. d) Example of ODMR spectra of the same ND in water and subsequently in BSA solution (left: bare ND, right: functionalized ND, normalized to the same contrast, 37°C). c) Illustration of the ND model. On the left side, a ND is represented with the NVs distributed assuming a constant density within the particle. Because of charges close to the shallowest NVs, an electrical field is produced and decreases as the distance from the surface increases (see the right side). The electrical field reaches a minimum that we define as a bulk electrical field. The magnitude and orientation of the bulk and surface electrical fields are fitting parameters and the NVs' orientations are assumed to be distributed evenly into the four possible directions they can take in the diamond lattice (for more details see Experiment Section). e) Fitting example of experimental data using the ND model (solid lines represent the ODMR spectra of the concentric shells, the dotted line represents the sum of the spectra and blue dots are experimental data).

**Figure 3:**
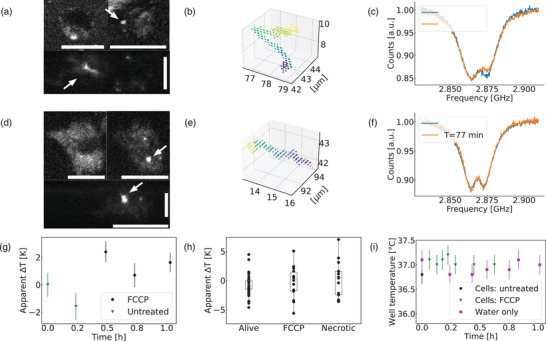
CW‐ODMR measurements in macrophages during FCCP treatment and necrosis (2.4 μ m). a) Primary human macrophage with an internalized and bare ND (termed ND‐B) indicated with an arrow (top: two XY section at different Z; bottom: Z section, see another example of confocal picture with intracellular ND in Figure S.11, Supporting Information). b) Single‐particle tracking localizations during a 6 min CW‐ODMR measurement on the intracellular ND‐B shown in the previous picture (the purple point is the starting position). c) ODMR spectra of the unfunctionalized ND‐B at two different time points inside the cell. d) Primary human macrophage with an internalized functionalized ND (termed ND‐F) indicated with an arrow (top: two XY sections at different Z; bottom: Z section). e) Single‐particle tracking localizations during a 5 min CW‐ODMR measurement on the intracellular ND‐F. f) ODMR spectra of the functionalized ND‐F at two different time points inside the cell. g) Example of increased apparent temperature readings caused by FCCP addition (error bar from the two Lorentzian fitting see Experiment Section). h) Box plot of apparent temperature readings for unfunctionalized NDs. The delta is relative to the first measurement done inside the cell (untreated). N = 6 cells for necrosis (untreated cells and FCCP treated cell included), N = 4 cells for FCCP (5–9 measurements per cell). The necrotic condition includes treated and untreated cells. Each point represents an intracellular measurement, the box is from the first to the third quartile with the orange line being the median. The whisker lengths are 1.5 times the inter‐quartile range. Durations of the CW‐ODMR measurements were typically 5–10 min long depending on the ND fluorescence counts. i) Time‐trace of temperature readings of the well in which cells are imaged. One trace is with macrophages during one of the FCCP experiment compiled in the boxplot, the other trace is only with water to measure the SD of the well temperature overtime: SD = 0.18 K. Therefore, the value of 0.18 K was used for error bars. The addition of FCCP did not cause a significant increase of temperature (within our error bars). For all confocal images: 513 nm excitation, 2 μW before the objective, horizontal and vertical scale bars: 10 μ m.

We first used our custom‐made confocal setup to measure the CW‐ODMR spectra of NDs in water and in protein solutions (see Experiment Section). Indeed, human cells are known to contain elevated protein concentrations (200–300 g L^−1^) but the effect of proteins on ND thermometry has not been studied;^[^ ^27^ ^]^ therefore, we investigated the impact of a highly concentrated protein environment on the ODMR spectra of our high‐pressure high‐temperature (HPHT) NDs. We chose to use bovine serum albumin (BSA) as it remains soluble at high concentration and is easily available with little contaminants (molecular biology grade). When immersed in a 300 g L^−1^ (BSA) solution, the full width at half maximum (FWHM) of the ODMR spectra typically increases by ≈20 % (N = 9) compared to water as shown in **Figure** **2**b,d (more examples in Figure S.4, Supporting Information). Interestingly, the relative depth of the dips can also change (Figure S.4g, Supporting Information) and similar spectral changes are found with intracellular NDs over time (**Figure** **3**c). We made sure laser and microwave excitation did not cause any heating (Figure S.8, Supporting Information) and then compared the ODMR temperature readings of the same NDs in water and in BSA while keeping the temperature of the objective and the stage constant (well temperature at 310.15 K or 37°C +/–0.18 K see Figure 3i). When measuring the apparent temperature difference reported by the NDs from water to BSA, the deltas have a standard deviation (SD) of 3.0 K (8 NDs, 8 measurements) while the temperature reading of 3 NDs in water over 5 h showed a SD of 1.8 (27 measurements).

We first thought that the charge state of the NV center could be changed upon BSA addition as the negative form of the NV center is known to dynamically switch to its neutral form. The neutral NV center has a fluorescence spectrum 60 nm blue‐shifted, but its electronic structure does not allow for ODMR.^[^ ^13^, ^28^ ^]^ Studies have shown that charge state conversion of shallow NVs (10 nm deep) can happen when NDs experience changes in surface termination or media.^[^ ^29^, ^30^ ^]^ Consequently, if shallow NVs would switch to a different charge state in BSA, we could be collecting the ODMR signal from a different NV population than in water. We could reject this assumption as no change of the fluorescence spectra was found when NDs were exposed to BSA after being in water (Figure S.1, Supporting Information). Thus, we hypothesized that electrical fields surrounding shallow NVs are responsible for this noise and the spectral changes, as shallow NVs may experience a fluctuating electrical field caused by surface changes. Electrical fields are known to cause a splitting and a relatively small shift of the ZFS that could explain the spectral broadening and the variation in the thermometry (Figure 2d).^[^ ^31^, ^32^ ^]^

To confirm this hypothesis, we covalently functionalized the same type of NDs with zwitterionic dipeptide moieties buffering the shallow NVs from direct exposure to electrical field fluctuations caused by surface changes (Figure 2a). The zwitterionic dipeptide moieties were shown to not only stabilize the nanoparticles in physiological environments but also to prevent non‐specific interactions with serum proteins.^[^ ^25^ ^]^ The functionalized NDs exhibited more robust ODMR spectra when immersed in a BSA solution as the FWHM decreases by ≈7% on average (N = 7, Figure 2b,d). Furthermore, the SD of the temperature reading (from water to BSA) is also improved for the functionalized NDs as it lowers down to less than 1 K (N = 3) compared to 3 K (N = 8) for the bare NDs (Figure 2b). Finally, we found that the charge of functionalized NDs remains negative in the presence of salts or a molecular crowding agent, and across typical intracellular pH values (pH 4 to 9, see zeta potential values in Table S1, Supporting Information). The zeta potential measurements are consistent with the fact that the ODMR spectra of the functionalized NDs changed very little between pH 4.5 and 9 (see all spectra in Figure S.10, Supporting Information).

In order to be able to better quantify the changes in the electrical field, a simplistic model of the ND was created to describe how surface charges affect the ODMR spectra of the particle. Assuming a spherical shape of the NDs and a field produced by the surface charges, we derived an expression of the electrical field as a function of the radius (see Equation E.7, Supporting Information). The simulation adds then to this field a constant electrical field produced by charges within the diamond (bulk field) and applies this summed field to all homogeneously distributed NVs inside the particle (Figure 2c). We can show with this simulation that the ODMR spectra broadens when the electric field produced by surface charges increases, also the asymmetry of the two dips can be explained by specific orientations of the NVs relative to the electrical field (Figure 2e).

The model can also be used to fit our experimental data with a view to measuring the average electrical field sensed by the shallowest NVs (termed $∥\vec{E_{s1}}∥$) and how it changes in a BSA solution. We found a typical magnitude of 8 × 10^5^ V cm^−1^ for $∥\vec{E_{s1}}∥$ in water with an increase of ≈14 % when BSA is added (see Supporting Information for details).

### 2.2 Continuous Wave ODMR Inside Macrophages

NDs were then introduced into primary macrophages in order to perform nanothermometry and explore how intracellular media affect the ND's surface electrical field. After incubating the cell with unfunctionalized NDs, the NDs were found free in the cytoplasm or in endosomes depending on when NDs have entered the cell as ND endosome escape has been reported in macrophages (Figure 3a; Figure S.11, Supporting Information).^[^ ^33^, ^34^ ^]^

The low intracellular density of NDs (1 or 2 NDs per cell) combined with single‐particle tracking, fluorescence counts and ODMR spectra allowed us to track the same NDs for hours despite cellular migration and ND movement.^[^ ^35^ ^]^ Interestingly, as the ND moved inside the cell, its CW‐ODMR spectrum showed the same changes (Figure 3c) that were described in the previous section (FWHM and relative depth of the dips, Figure 2d; Figure S.4, Supporting Information). The spectral alterations were also correlated to large variations (up to 4.6 K) in temperature readings over the course of a few hours without any cell treatment (Figure 3h).

To stimulate single cell thermogenesis and attempt to measure it, cells were treated with carbonyl cyanide‐*p*‐trifluoromethoxyphenylhydrazone (FCCP), a compound able to disrupt mitochondrial membrane potentials by uncoupling the proton gradient from ATP synthesis. Like the uncoupling protein 1 (UCP1), FCCP triggers an increased oxidative activity in cells and a higher heat generation rate is expected.^[^ ^36^ ^]^ The addition of FCCP caused a slight apparent increase of temperature (i.e. decrease of ZFS, see example Figure 3g) in macrophages but this increase was hardly significant as readings before FCCP treatment had already a large standard variation (1.9 K, 4 cells, 5–9 measurements per cell see Figure 3h). This change of the ZFS could be interpreted as a heating caused by a higher mitochondrial activity as previously reported.^[^ ^4^, ^37^ ^]^ In our case, the apparent temperature increase is more likely to be only caused by changes in the surface electrical field. Furthermore, more than 20 min after cells were killed (necrotic) using high laser power, no clear temperature decrease was observed (Figure 3h). It is worth mentioning that the ODMR spectra of NDs immobilized on the glass slide 12 micrometers away from the cell did not exhibit ODMR spectral changes and provided stable temperature readings during the experiment (Figure S.12, Supporting Information). Likewise, the temperature of the well measured with a thermocouple probe did not show any significant increase of temperature following FCCP addition (within our error bar of 180 mK see Figure 3i).

To test whether surface electrical fields are the cause of the noisy ZFS measurements, we performed the same experiment with functionalized NDs. This time NDs showed the same ODMR spectra for more than 1 h (Figure 3f) and reported no increase in temperature upon FCCP addition while exhibiting smaller SD of the temperature readings (functionalized NDs: 1.25 K, 9 measurements, 1 cell for untreated and FCCP condition see Figure S.14 (Supporting Information), versus a SD of 2.25 K, 44 measurements, 4 cells for bare NDs). The fact that the temperature readings were stabilized after functionalization supports our interpretation that electrical field fluctuations produced by intracellular proteins or other cellular components (e.g. salts) are causing spectral changes and erroneous temperature readings.

Using the model described previously, the variation of the electrical field sensed by the shallowest NVs ($∥\vec{E_{s1}}∥$)) was investigated for the three different cellular conditions (untreated, FCCP and necrotic). Fluctuations of the $∥\vec{E_{s1}}∥$ increased for dead cells as we would expect since the interaction of the ND's surface with the environment is greatly affected after necrosis (Figure S.13, Supporting Information).

### 2.3 Pulsed Measurement

The precision of the measurement was still on the order of 1 K (fit error for single Lorentzian dip) even when using functionalized NDs, so we turned to pulsed measurements to improve precision. One approach is to apply a Ramsey‐based temperature measurement protocol, termed D‐Ramsey, which has been used to demonstrate very high temperature resolution with NDs (down to 1 mK accuracy).^[^ ^18^ ^]^ It requires the application of a bias magnetic field to split the energy levels of the ±1 states, as shown in Figure 1. Then, the protocol applies selective microwave pulses on the transitions |0〉↔| − 1〉 and |0〉↔| + 1〉 to remove the effect of noise due to small magnetic field fluctuations.^[^ ^18^ ^]^ This allows for high″sensitivity measurement of the transition frequency between the zero‐field splitting *D* (dependent on temperature). However, this D‐Ramsey protocol is challenging to implement inside cells as it typically requires single NVs and a constant energy difference between the |0〉 and | ± 1〉 transitions. The pulsed sequence necessitates to fix the NV position relative to a bias magnetic field during the entire measurement, which is very challenging inside cells as both the cells and NDs typically move (Figure 3b).

**Figure 4:**
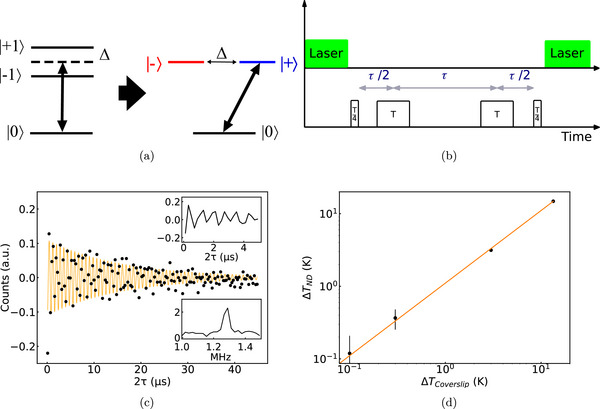
Pulsed nanothermometry. a, Scheme of “thermal echo” (TE). When the 3‐level system (| ± 1〉 and |0〉) interacts with a linearly polarized microwave field, the coherent superposition states $|\pm \rangle =\left(|+1\rangle \pm |-1\rangle \right)/\sqrt{2}$, become the eigenstates of the system. Magnetic, electrical fields, and hyperfine coupling to the inherent nitrogen nucleus lead to an additional splitting of the spin states. These effects can typically be combined as an effective detuning Δ that causes splitting between | ± 1〉 and corresponds to coupling between | ± 〉.^[^ ^38^ ^]^ We prepare the system in state |0〉 by illumination with a laser pulse. Under continuous microwave field with a frequency ω_*c*_ = *D* = (2π)2.87 GHz, we observe oscillations of the population of state |0〉 with a frequency 2$\bar{\Omega }$ due to transitions between states |0〉 and | + 〉. If Δ ≠ 0 there are also oscillations with frequency $\bar{\Omega }=\sqrt{\Omega^{2}+\Delta^{2}}$ due to the coupling to | − 〉.^[^ ^38^ ^]^ b, If Ω > Δ, it is possible to perform a TE by creating a superposition between |0〉 and | + 〉 with a pulse with duration *T*/4 (taking $T=\pi /\bar{\Omega }$, in this experiment T = 43 ns see Experiment Section for more details). Applying pulses of duration *T* takes | + 〉 → −| + 〉 and back, thus refocusing the effect of Δ. During the free evolution, the coherence accumulates a phase, which depends on the zero‐field splitting *D* and thus on the temperature. Finally, the coherence is mapped back to the populations by a *T*/4 pulse, allowing the detection of *D*. The sequence can be implemented with rectangular pulses or with RAP pulses (see Experiment Section). c, TE oscillations of a ND (2‐4 NVs) obtained from a differential measurement that corrects for laser noise and NV charge state fluctuations. When the frequency of the microwave pulses is different from D (in this case detuned by (2π)2.59MHz), oscillations corresponding to the frequency difference can be observed (in our case it is undersampled oscillations corresponding to half of the detuning: 1.28 MHz). Top inset is a zoom of the oscillations and the bottom one is the FFT with amplitude as y axis). If NVs have significant differences in D multiple peaks may be observed (see Figure S.21, Supporting Information). d, Comparison of the TE reading with the temperature of the coverslip on which the ND is immobilized. The temperature dependence of D was ≈10% larger than the –74 kHz K^−1^ originally reported as the best fit in the Figure is *y* = 1.10*x*. Such differences have already been reported in NDs.^[^ ^12^, ^14^ ^]^ Black dots: experimental data; orange line: fit.

To address this issue, we applied a pulsed protocol, shown in **Figure** **4**a, that does not require a bias magnetic field and can be applied even when the ±1 states are degenerate.^[^ ^38^ ^]^ Specifically, we use a sequence of pulses to drive transitions between |0〉 and $\left|+\right\rangle =\left(|1\right\rangle +|-1\rangle )/\sqrt{2}$ states and refocus magnetic noise, similarly to low field dynamical decoupling in Vetter et al.^[^ ^38^ ^]^ We label this approach (see Figure 4a) a “thermal echo” (TE) to distinguish from the D‐Ramsey sequence in Neumann et al. as measuring *D* allows for efficiently detecting temperature.^[^ ^18^ ^]^ The TE protocol first creates a coherent superposition between states |0〉 and | + 〉, followed by two pulses to refocus magnetic noise. The accumulated phase of the coherence depends on *D* and the estimation precision is limited by *T*_2_ of the decoupling sequence (see Supporting Information). Then, a readout pulse microwave pulse maps the coherence back to the populations of |0〉 and | + 〉, which are then detected by laser illumination and state‐dependent fluorescence. We note that in some experiments we replaced the two rectangular refocusing pulses with rapid adiabatic passages (RAP) as they are robust against variations of Rabi frequency, which can be useful for NV ensembles (see Experiemental Section).^[^ ^39^, ^40^ ^]^

After testing the TE with and without RAP on single NVs inside bulk diamond, we applied TE on the 100 nm HPHT NDs used in the previous section. The coherence time of these particles was too low (T2* <500 ns) and we had to use high purity NDs synthesized by chemical vapor deposition (CVD). Because of their low nitrogen content (0.15 ppm) and ^12^C enrichment, our CVD NDs have remarkable spin properties ($T_{2}^{*}$ > 2 μ s and *T*_2_ Hahn echo > 20 μ s) at the expense of fewer NVs per NDs (1–10 NV/ND versus 100 NV/ND for the HPHT NDs).^[^ ^41^ ^]^ With an improved *T*_2_, the implementation of the TE on CVD NDs was successful, and we therefore performed thermometry on a ND immobilized on a glass slide. As we increased the temperature of the stage, the temperature could be very well measured with the NDs with precision down to 80 mK in a few minutes acquisition (Figure 4d). By fitting the decaying oscillations, we obtain a sensitivity of 1.1 $K/\sqrt{Hz}$ and a maximum precision of 52 mK (Figure 4c; Figure S.17, Supporting Information). Similar results were obtained using RAP pulses instead of rectangular pulses.

Before intracellular sensing with CVD NDs, we made sure the ODMR spectra and the fast‐Fourier transform (FFT) of the TE signal were not affected by proteins. Once in the BSA solution, the ODMR spectra of CVD NDs did not significantly change (Figure S.18, Supporting Information); similarly, peaks from the FFT did not shift upon BSA addition although we did observe the disappearance of peaks in some cases suggesting a possible loss of coherence time for shallow NVs. We tried to mitigate this effect with dipeptide functionalization but found that the contrast of pulsed measurements was decreased. We do not know the reasons for this loss of contrast and can only speculate that the higher peak laser power required for pulsed measurement might interact with the dipeptide functionalization.^[^ ^42^ ^]^ Consequently, we decided to use bare CVD NDs for the intracellular experiments.

**Figure 5:**
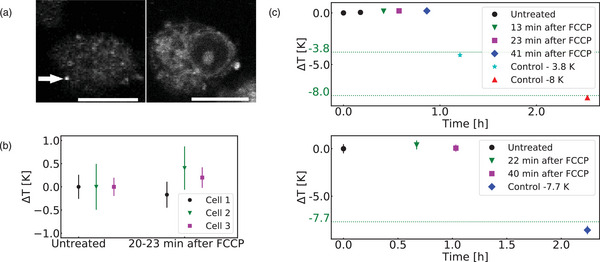
Pulsed nanothermometry inside living cells. a) Confocal image of the macrophage used for the nanothermometry measurement. Here, the CVD ND happens to be located on the lower part of the cell (left picture being the lower part of the cell, the CVD ND is marked by an arrow, 513 nm excitation, 2–4 μW before the objective, scale bar 10 µm). b) Comparison of the temperature measurement before and after FCCP for 3 cells (2.4 μ m). c) Time‐trace of temperature measurements (RAP TE) for cell 1 and 3. Here, the temperature of the stage was intentionally lowered to further verify the ND's response. The green lines show drops in the well's temperature measured with the thermocouple. Temperature is stable before and after FCCP addition (within our error bars, SD of all the measurements = 150 mK) and our ND readings are also consistent with the thermocouple measurements when the well temperature is lowered. The difference between the thermocouple and the ND readings (0.2–0.8 K) might be due to temperature gradients between the ND and the thermocouple probe (1 cm apart).

Even with a reduced signal‐to‐noise ratio caused by cell autofluorescence and ND displacement, we could observe in some NDs the oscillations produced by the TE sequence with RAP to measure temperature inside the cell (Figure S.20, Supporting Information). As shown in **Figure** **5**c (top, see FFTs in Figure S. 21, Supporting Information), the ND temperature did not fluctuate for more than 40 min (SD of the temperature: 100 mK) despite the addition of FCCP into the media. Nonetheless, the functioning of the ND sensor was demonstrated after 1 h when the temperature of the stage was lowered by 3.8 and 8 K successively (ND reading –4.1 and –8.2 K). A similar time‐trace with such additional control was generated for another cell (Figure 5c bottom). We also compared the temperature before and 20 min after FCCP addition for a third cell and found no significant temperature change upon FCCP addition (within our error bars: 150–500 mK depending on the cell, see Figure 5b).

## 3 Discussion

An important artifact is reported on unfunctionalized NDs: high protein concentrations strongly affect the electrical field surrounding shallow NVs which changes the FWHM and shape (i.e. dip asymmetry) of the ODMR spectra. The strong electrical field on the surface will not only increase the splitting of the ODMR spectra but will shift the spectrum thus causing apparent temperature fluctuations. This causes significant noise in thermometry and could lead to misinterpretation as shown in the intracellular measurements (Figure 3). More robust techniques such as the 4‐point method or the frequency‐jump modulation do not correct for this effect.^[^ ^12^, ^17^, ^43^ ^]^ We show that with dipeptide functionalization, the SD of thermometry can be reduced. Remarkably, a recent report also revealed the same effect of electrical fields on ND temperature readings, while using a more complex Hamiltonian to explain the shift of the ODMR spectra inside macrophages during inflammatory response.^[^ ^44^ ^]^ Like in our study, they were able to mitigate such effects by changing the ND surface but used a different chemical approach (silica coating).

Even with the dipeptide moieties, the noise (inside cells and immobilized on the glass slide) could not be reduced to the 100 mK that was reached using TE with CVD NDs. The improvement of the sensitivity is likely due to the superiority of pulsed measurement over CW‐ODMR combined with the great spin properties of our CVD NDs. Indeed, the uncertainty of the Lorentzian fit for the best CW‐ODMR spectrum of our CVD ND shown in Figure S.18 (Supporting Information) is ≈800 mK, which is not as good as the sine fitting error of the echo signal (52–100 mK) for similar acquisition time (5–10 min).

We developed a model to understand better this artifact and found that strong electrical fields at the ND's surface (average of 8 × 10^5^ V cm^−1^) can cause spectral broadening. Point charges in diamond close to the NV center could be responsible for such fields as previously reported.^[^ ^45^, ^46^ ^]^ We also found that the distribution/orientation of electrical fields can explain the change of depth between the two dips. Usually, this asymmetry is explained by the orientation of the linearly polarized microwave field with respect to the NVs.^[^ ^47^ ^]^ Although this phenomenon should not be disregarded, we believe it does not play a significant role in our HPHT NDs. Indeed, the ODMR spectra of diffusing and rotating NDs in glycerol showed the same spectral shape at multiple time points (Figure S.22, Supporting Information).

The perspective we took on the interpretation of low field CW‐ODMR spectra could open new analytical methods to investigate colloidal science but also measure the change in electrical field when NDs or bulk diamond are interacting with biomolecules with a view to carrying out molecule fingerprinting or sequencing for instance. To do so, improvements in our model should be carried out, such as taking into account the irregularity of the NDs shapes and the dynamic behavior of charges. More importantly, a more precise understanding of how molecules such as proteins on the ND surface interact with NV physics needs to be reached.

While the effect of elevated protein concentration has not been studied before, previous research did investigate the effect of different solutions (e.g. high salts, glycerol, ethanol, 4–10 pH buffered solution) on NV's spin properties.^[^ ^48^, ^49^ ^]^ Fujiwara et al. reported that pH can add noise to the measurement of the ZFS (parameter D) as ODMR spectra and fluorescence counts were affected.^[^ ^49^ ^]^ If pH can modify the ODMR spectra, it is likely that high concentrations of proteins and other factors such as salts, solvent polarity or redoxactive environments have comparable impacts. Thus, the phenomenon we report can have important consequences not only for temperature sensing but also for other types of ND biosensing approaches such as *T*_1_ relaxometry for radical sensing or ND orientation measurements.^[^ ^23^, ^35^, ^50^ ^]^

We hypothesize that the mechanism for this electrical noise might rely on non‐photoactive defects like substitutional nitrogen or vacancies, that would exchange charges with the surface as previously discussed.^[^ ^51^ ^]^ This process would also explain why we do not observe such strong effect on high purity CVD NDs that did not undergo electron irradiation. Another explanation would be the lower number of NV/ND in the CVD NDs compared to the HPHT NDs that would make the NVs less likely to be close to the surface.

Once we characterized the behavior of our ND sensor in complex environments, we could implement an advanced quantum sensing protocol to reach remarkable sensitivity outside and inside cells (100 mK precision). Higher sensitivity could be achieved by measuring the fluorescence on one point of the decaying oscillations as showed by Kucsko et al. but this approach has limited dynamic range and we expected it to be difficult to use intracellularly because of cell autofluorescence.^[^ ^17^ ^]^

To our knowledge, the work presented here is the first nanothermometry report to reach the intracellular target resolution (100 mK) required to study new biological processes such as epilepsy and cardiovascular accidents.^[^ ^1^ ^]^ Furthermore, regarding the controversial single‐cell thermogenesis findings, our TE readings are the most precise attempt to measure heat produced by individual cells, and yet we did not detect temperature fluctuations upon FCCP treatment unlike previous reports. Indeed, multiple nanothermometry reports using different types of approaches described a 1–2°C increase of the cell body (mostly cytoplasm) once FCCP is added.^[^ ^4^, ^37^, ^52^, ^53^ ^]^ As temperature differences of a few degrees inside single cells is controversial we can interpret our results in the two conflicting interpretations. On one hand, our results are consistent with previous theoretical considerations and microcalorimetry measurements.^[^ ^8^, ^54^ ^]^ On the other side, if such large gradients exist, our finding may not be fully comparable to the previous studies since we used primary macrophages while previous reports used cell lines (COS7 or Hela cells). Primary macrophages are a suitable model for our study because they are more representative of normal in vivo conditions and would allow us to investigate more realistically inflammatory processes. Additionally, thermogenic response of primary macrophages (measured by their respiration rate in Figures S.15 and S.16, Supporting Information) is expected to be greater than in Hela cells. Nonetheless, cells are remarkably complex and we cannot exclude the fact that other physicochemical processes could explain the difference between our findings and previous reports. We note that our NDs did not specifically target the mitochondria, that is why further studies are required to fully reconcile both interpretations. For instance, future investigations could perform a high‐throughput TE study with mitochondria targeting in conjunction with the nanothermometers that measured the temperature increase upon FCCP treatment. It is also worth mentioning that it is not possible to know if the artifact reported here in NV thermometry is also applicable to the other varied nanothermometers used in previous studies as the physicochemical principles of the measurement are very different from NV sensing.

Intracellular TE is limited to CVD NDs with long coherence properties ($T_{2}^{*}$ > 2 μ s and *T*_2_ Hahn echo > 20 μ s). The protocol producing our CVD NDs gives us enough amount for thousands of TE experiments but future improvements in HPHT NDs synthesis could make TE experiment more easily accessible. The sensitivity of TE could be improved by investigating the remaining noise sources when performing intracellular TE such as cell autofluorescence, ND displacement and other physicochemical process that may occur inside the cell. Because not every intracellular ND shows a TE signal, the throughput of our technique could be enhanced by brighter NDs and an adjusted functionalization protecting NVs while maintaining good ODMR contrast. Finally, the implementation of wide‐field quantum sensing instead of confocal microscopy would dramatically increase throughput and make ND pulsed measurement a technique that could be routinely used to investigate heat diffusion inside living cells.

## 4 Conclusion

In conclusion, we present here strong coupling of NV ODMR spectra with the ND surface and show that it may introduce artifacts in temperature sensing. Careful controls on this surface coupling need to be done when performing intracellular ND‐based sensing like nanothermometry. By expanding our understanding of this interaction, we opened new sensing possibilities that could, for instance, be helpful to explore open questions in colloidal science such as the magnitude and spatial distribution of electrical fields at the interface of nanoparticles.^[^ ^55^ ^]^ Moreover, we unlocked the potential of ND pulsed measurements for intracellular investigation by combining the use of high‐purity NDs with robust coherent control. We achieved a 50–100 mK precision that we leveraged to investigate the controversial and important question of single‐cell thermodynamics. We find that the interpretation of the heat diffusion law is consistent with our results as we did not find temperature changes upon metabolic stimulation. Nonetheless, nanoscale intracellular thermodynamics is a rich and complex topic. As further nanothermometry studies will allow us to fully understand the discrepancy in the literature and the scale of intracellular gradients, the great precision of ND TE is likely to play an important role. It would be also interesting to perform nanothermometry measurement in a multicellular context to see how gradients can locally affect the functioning of organs or tissues. With long coherence properties and robust coherent control, more diverse quantum sensing protocols can now be used inside living cells in order to study a wide range of biophysical and biochemical phenomena.

## 5 Experimental Section

### Nanodiamonds and Functionalization

**HPHT NDs**: The 100 nm HPHT NDs containing ≈100 NVs per particle were prepared as described previously.^[^ ^56^ ^]^ Particles from Microdiamant (Pureon ref.: MSY0.1) were electron irradiated (0.5 × 10^18^ cm^−2^) and simultaneously heated to 800 °C. After irradiation, NDs were subjected to air oxidation at 620 °C for 5 h to remove the graphitic residues from the surface.

**CVD NDs**: CVD NDs were obtained as described previously from isotopically purified polycrystalline ball‐milled NDs (N^0^_S_ concentration of 0.15 ± 0.02 ppm). The starting CVD diamond material from which the NDs were milled is from Element Six.^[^ ^41^ ^]^ The sample was not irradiated nor annealed. Si_3_N_4_ balls to avoid magnetic contaminants from steel ball milling were used. After milling, the sample was acid cleaned in H_3_PO_4_ and then cleaned in NaOH, to remove the Si_3_N_4_ contaminants. The nanodiamonds were then annealed in an air atmosphere, dispersed in water, and centrifuged at a relative centrifugal force of 40 × 10^3^ g. CVD NDs showed a size typically smaller than 100 nm as shown in March et al.^[^ ^41^ ^]^

**Functionalization**: In brief, azide functionalized ND‐O‐N_3_ (for details see Supporting Information) was washed twice with dimethylformamide (52 k rpm, 30 min) before redispersing it in 10 mL of degassed dimethylformamide. The dispersion was degassed under nitrogen atmosphere by bubbling nitrogen through it and simultaneously sonicating for 15 min. Then, 13.3 mg of copper(II) sulfate (0.08 mmol) were added and the degassing procedure was repeated. Next, 33.3 mg of sodium ascorbate (0.17 mmol) were added and the dispersion was stirred for 1 h before addition of 20.0 mg of Tyr(*O*‐propargyl)‐Leu (0.06 mmol). The dispersion was stirred under nitrogen atmosphere and at room temperature for 5 days. Afterward, the particles were ultracentrifuged (52 k rpm, 30 min) and washed consecutively with dimethylformamide (3x), acetone (3x), ethylenediaminetetraacetic acid (EDTA, 0.1 m) solution (3x) and doubly distilled water (3x) to yield ND‐O‐Tyr‐Leu. FT‐IR (DRIFTS): $\tilde{\nu }$ = 3444 (br, ν(O‐H)), 2962 (m, ν(C‐H)), 2876 (w, ν(C‐H)), 1643 (m, ν(C = O), amide I), 1509 (w, ν(C = C_arom_)), 1458 (w), 1374 (s, δ(O‐H)), 1263 (m), 1217 (w, ν(C‐O)), 1179 (w), 1098 (m, ν(C‐O)), 942 (w), 811 (m, δ(C‐H_1,4‐arom_)), 711 (w), 685 (w), 529 (w) cm^−1^. Zeta potential: −21.0 ± 0.25 mV (doubly distilled water, intrinsic pH = 6.5). Particle size (DLS): 10 % 92.6 ± 6.61 nm, 50 % 154 ± 3.00 nm, 90 % 244 ± 10.6 nm (doubly distilled water). The synthesis of the precursor as well as additional analytical data can be found in the Figure S.2 (Supporting Information).

### Confocal Quantum Sensing Setup

All the quantum sensing measurements (immobilized NDs and inside cells) were performed on a home‐built confocal microscope as described in Wu et al. with the following changes (see Figure S.7, Supporting Information).^[^ ^21^ ^]^ A 40X oil‐immersion objective with a 1.4 NA (Olympus, UPLXAPO40XO) was used. Laser pulses were created with an arbitrary waveform generator (AWG) 70001A from Tektronix and a 513 nm laser from Toptica Photonics (Ibeam‐smart 515.S‐15133). The laser beam was filtered by a 513 nm bandpass filter (HQ515/20M, Chroma), then a lambda half plate (AHWP10M‐600, Thorlabs) followed by a polarizing beamsplitter (PBS121, Thorlabs) was implemented to further control the laser power. A piezo stage (P‐562.3CD) from Physik Instrumente was used for the objective' s nanopositioning. Fluorescence was collected by an Excelitas avalanche photodiode (SPCM‐AQRH 13) with a 590 nm long pass filter (ET590lp, Chroma). Pulse averaging was performed with a FAST ComTec time tagger (MCS6A1T2) and a National Instruments card (6343) allowed us to manage the analogue/digital interfacing between the computer and the microscope. Fluorescence spectra were collected by a Kymera spectrometer from Oxford instruments (Camera iDus 416). A 16 W microwave amplifier (ZHL‐16W‐43‐S+, Mini‐circuits) was used to amplify microwave pulses.

For temperature control, an objective heater (Objektivheizer 2000) and heated insert (P Lab‐TekTM S1) from Pecon was used. According to the manufacturer the temperature accuracy of the heated components (not the well temperature) is 0.1 K. A thermocouple thermometer (Voltcraft K102) was also used to measure directly the temperature of the well when necessary. According of the manufacturer the accuracy is 1 K but the accuracy was found to be much lower (see details in the Statistical Analysis). CO_2_ and humidity control was also performed with a CO2‐Controller 2000 and Humidity System 2000 from the same company respectively. The entire experiment was controlled by a customizable open source software: Qudi.^[^ ^57^ ^]^

### Cell Preparation and Nanodiamond Treatment

Peripheral blood mononuclear cells (PBMCs) were extracted from the peripheral blood of healthy donors (sourced from the German Red Cross blood donation center at the University Hospital Ulm, written consent from all participants or next of kin was obtained prior to the research) using density gradient centrifugation with Ficoll Paque (GE Healthcare, Chalfont St. Giles, UK). A total of 21 × 10^7^ PBMCs were incubated in a plastic cell culture flask for 60–90 min in macrophage serum–free medium (MSFM) to allow adherence. Non‐adherent cells (lymphocytes) were removed through washing. In contrast, adherent cells (monocytes) were cultured for six days in MSFM containing 10 ng mL^−1^ granulocyte‐macrophage colony‐stimulating factor (GM‐CSF, Miltenyi, Bergisch Gladbach, Germany) to generate monocyte‐derived macrophages. These Macrophages were harvested on day 6 using PBS‐EDTA, counted using a Neubauer Chamber, and seeded in a glass vial with NDs at a concentration of 900 000 macrophages per milliliter. For HPHT NDs, a concentration of ≈1 pm (1 mg L^−1^) and 50 pm (50 mg L^−1^) for CVD NDs. ND incubation time was 2 h or overnight depending on the endocytosis rate of the macrophages (due to donor‐donor variability). Optimal concentrations of FCCP (2.4 μ m) were titrated by extracellular flux analysis using a plate‐based respirometer (XFe96, Agilent Biotechnologies). Primary human macrophages were plated with a density of 5 x 10^4^ cells per well in XF RPMI medium supplemented with 10 mm glucose, 1 mm pyruvate, and 2 mm glutamine into XF96 cell culture plates. Increasing concentrations of FCCP (Sigma‐Aldrich) were injected until full substrate oxidation capacity was reached. Non‐mitochondrial respiration was determined by injecting 0.5 mm antimycin A and 0.5 mm rotenone (ETC inhibitors, Sigma–Aldrich). The 2.4 μ m for primary macrophages was the optimal concentration with and without ND incubation (2 h, HPHT NDs at 0.1 pm). Cell viability was checked at a single cell level based on the morphology, NDs displacement and the cell autofluorescence level (< 2 × 10^5^ counts per second at 2 μW laser power before the objective). Trypan blue viability test confirmed that highly autofluorescent cells have lost membrane integrity.

### Quantum Sensing Measurement and Imaging

Measurements on immobilized NDs were done on drop casted ND solution with a concentration of 30–60 pm (30–60 mg L^−1^). Silicon wells forming 30–50 μL wells (Grace Bio‐Labs) were used to study the effect of water/BSA on the ODMR spectra and 300–500 μL wells for intracellular measurements. The BSA solution is simply BSA powder (Sigma Aldrich ref.:A9418) freshly mixed with Milli‐Q water. A gold wire soldered (20 μm thick) with indium to a custom made printed board circuit was used to deliver microwave inside the wells. All the measurements were done without any bias magnetic field. Durations of the CW‐ODMR and pulsed measurements were typically 5–15 min long depending on the signal‐to‐noise ratio. Shorter acquisitions were preferred in intracellular measurements to limit the phototoxicity caused by the laser. Imaging of NDs inside cells was typically done with 2–4 μW 513 nm excitation using a 1000 Hz scanner frequency. Cell body will usually display a fluorescence of 50 to 150 × 10^3^ counts per second while HPHT NDs will exhibit 0.4 to 2 × 10^6^ counts per second.

**CW‐ODMR**: NDs were preselected for temperature measurement based on their ODMR spectra (good double Lorentzian fitting). A laser power of 2 μW was used with 30 mV amplitude on the AWG for the microwave signal. Cells were killed with > 100 μW excitation for 20–30 min (until the cell reduced in size or exploded). Cell death was also confirmed with no ND displacement and cell autofluorescence exceeding 2 × 10^5^ counts per second at 2 μW. The error bar for temperature sensing was calculated based on the error of the Lorentzian fit (see Statistical Analysis for more details).

**Pulsed measurements**: NDs were preselected based on their Rabi period (corresponding to Rabi frequency varying between (2π)5 − 25 MHz, defined here as 2$\bar{\Omega }$) and ODMR contrast (15‐30%). A laser power of 10‐100 μW was used depending on the counts obtained from the CVD‐NDs (usually > 10^5^ counts per second) as intracellular measurements required to minimize phototoxicity. A ND filter was usually required to lower the fluorescence count before the APD. Laser pulses of 10 μs were used with a readout window of 200–500 ns. Shorter laser pulses were causing a local heating as the time between the microwave pulses was too short to let the heat from the microwave pulses dissipate. The parameters for RAP pulses were typically: detuning (amplitude) with $\tanh(t/\tilde{\tau })$ ($\text{sech}(t/\tilde{\tau })$) shape, similarly to Genov et al. with 10 MHz frequency detuning range and peak Rabi frequency $2\bar{\Omega }=\left(2\pi \right)5-25$ MHz.^[^ ^39^ ^]^ The refocusing was done once by two RAP pulses, each with duration of 100 ns. The pulses have a truncation ratio of 0.1, resulting in characteristic time $\tilde{\tau }=10$ ns. The first and last T/4 pulse were replaced with the first and second half of the RAP pulse respectively (duration 50 ns). The center of the frequency sweep range of the RAP pulses was 1–4 MHz lower than the ZFS measured on the CVD‐ND of interest. It was noted that due to the large variation of the Rabi frequency caused by ND/cellular movement, the adiabaticity condition for RAP is often not satisfied despite the optimization of experimental parameters. Future optimization of the pulses and sequences is expected to improve performance but goes beyond the scope of this work. If an undersampled frequency is observed in TE oscillations, a correcting factor needs to be applied to convert frequency to temperature.

The temperature of the well was constantly checked during the various experiments with a thermocouple probe. When opening the lid (to add the freshly prepared room temperature pre‐diluted DMEM FCCP solution), because the air of the room is colder (21°C), the temperature quickly drops by ≈3°. It was measured that it takes ≈2 min to go back to 37°C. Nanothermometry and thermocouple readings were taken only after the temperature equilibrates.

All the laser powers mentioned in the manuscript were measured in CW mode before the objective. The code for the surface potential CW‐ODMR spectra modeling/fitting and the RAP‐TE predefined method (on Qudi) are available on GitHub upon request (GitHub ID: Maabur).

### Statistical Analysis

**Thermocouple and well temperature**: The temperature of the well was regularly checked during all the experiments done here is solution (water, BSA solution and cells) using a thermocouple thermometer. It was found that once the heated components reach their target temperature, the temperature is stable within a SD of 0.18 K (N = 13 measurements, see an example of the time‐trace 3i). Therefore, the value of 0.18 K was used for error bars.

**Normalization**: For FWHM and temperature measurement raw CW‐ODMR spectra were used. CW‐ODMR spectra were normalized when displayed on the same plot. First, both spectra were normalized to 1 so the contrasts could be compared. Second, the spectrum with the lowest contrast was multiplied by a corrective factor and the baseline was adjusted to 1 by subtracting the spectrum with the difference it had with the baseline. **Error bars**: For the CW‐ODMR the spectrum is fitted with a double dip Lorentzian. The fit outputs 2 estimations of the dips position and with it, two uncertainties. The average of both positions was taken to determine the ZFS position. As an error bar for the ZFS (that will be used for the temperature error bar) the largest uncertainty was taken form the 2 errors produced by the fitting instead of taking the sum of both uncertainties. The sum was not taken because it led to an overestimation of the error as supported by the SD of 3 immobilized NDs in water over 5 h (27 measurements), it is also the case in the Figure S.8 (Supporting Information) and when comparing with the thermocouple controls (Figure 1).

**Outliers**: No outliers were excluded for the control experiments of S. 8. CW‐ODMR spectra were excluded from the FWHM boxplot 2b simply if the Lorentzian fitting failed. CW‐ODMR spectra were excluded from the temperature boxplot 2b analysis when the temperature readings had fitting error superior to 6° and/or a temperature delta of more than 9° as temperature fluctuation was not expected from this order. The same criteria were used to define outliers in the intracellular CW‐ODMR measurements. Regarding TE measurements (immobilized NDs and inside the cell) no outliers was found. For the noisy measurements (i.e. the intracellular TE see example Figure S.21, Supporting Information), fitting of the FFT using a Lorentzian was preferred over fitting the TE oscillations with sine functions.

**Software used for statistical analysis**: Fitting was performed with python packages (lmfit and scipy) directly or indirectly with Qudi whose fitting functions are built from the lmfit and scipy package.^[^ ^57^, ^58^, ^59^ ^]^

## Conflict of Interest

The authors declare no conflict of interest.

## Supporting information

Supporting Information

## Data Availability

The data that support the findings of this study are available in the supplementary material of this article.
