# Supplementary material for: Millikelvin Intracellular Nanothermometry with Nanodiamonds
Source: Adv Sci (Weinh). 2025 Sep 29;12(45):e11670. doi: 10.1002/advs.202511670 (PMC12677697; doi:10.1002/advs.202511670)
Supplement: Supplementary file 1 — Supporting Information [file ADVS-12-e11670-s001.pdf]

Supplemental Information to: millikelvin intracellular nanothermometry with nanodiamonds

Maabur Sow Jacky Mohnani Genko Genov Raphael Klevesath** Elisabeth Mayerhoefer** Yuliya Mindarava Rémi Blinder Soumen Mandal Fabien Clivaz Raúl B. Gonzalez Daniel Tews Christian Laube Wolfgang Knolle Amelie Jerlitschka Farid Mahfoud Oleg Rezinkin Mateja Prslja Yingke Wu Pamela Fischer-Posovszky Martin B. Plenio Susana F. Huelga Tanja Weil Anke Krueger Gavin W. Morley Oliver Williams Steffen Stenger Fedor Jelezko*

***These two authors contributed equally.*

Maabur Sow*, Genko Genov, Yuliya Mindarava, Rémi Blinder, Raúl B. Gonzalez, Farid Mahfoud, Oleg Rezinkin, Mateja Prslja, Fedor Jelezko*
Institute for Quantum Optics, Ulm University, D-89081 Ulm, Germany
*E-mail: maabur.sow@uni-ulm.de *E-mail: fedor.jelezko@uni-ulm.de

Jacky Mohnani, Raphael Klevesath**, Steffen Stenger
Institute for Medical Microbiology and Hygiene, Ulm University, 89081 Ulm, Germany

Elisabeth Mayerhoefer**, Amelie Jerlitschka, Anke Krueger
Institute of Organic Chemistry, University of Stuttgart, 70569 Stuttgart, Germany

Soumen Mandal, Oliver Williams
School of Physics and Astronomy, Cardiff University, Cardiff, United Kingdom

Fabien Clivaz, Martin B. Plenio, Susana F. Huelga
Institute for Theoretical Physics, Ulm University, D-89069 Ulm, Germany

Daniel Tews, Pamela Fischer-Posovszky
Department of Paediatrics and Adolescent Medicine, Ulm University, D-89075 Ulm, Germany

Christian Laube, Wolfgang Knolle
Leibniz Institute of Surface Engineering (IOM), 04318 Leipzig, Germany

Yingke Wu, Tanja Weil
Max Planck Institute for Polymer Research, 55128 Mainz, Germany

Anke Krueger
Center for Integrated Quantum Science and Technology, University of Stuttgart, 70569 Stuttgart, Germany

Gavin W. Morley
Department of Physics, University of Warwick, Coventry, United Kingdom

Contents

S.1 Functionalisation **3**
S.1.1 Nanodiamond characterisation and surface functionalisation 3
S.1.2 Methods for nanodiamond characterisation 3
S.1.3 Characterization of the fluorescent nanodiamond staring material model system 3
S.1.4 Reduction of the carboxylated starting material 3
S.1.5 Attachment of the aromatic azide linker 4
S.1.6 Conjugation with L-Tyr-L-Leu dipeptide by 'click' reaction 4

S.2 Derivation surface potential sensing **5**

S.3 Details on the Thermal Echo (TE) Scheme **11**

S.4 Setup and other controls **15**

S.1 Functionalisation

S.1.1 Nanodiamond characterisation and surface functionalisation

Two types of fluorescent NDs were treated in an identical procedure to ensure comparability between the results of the functionalisation. The HPHT highly fluorescent NDs eventually used for the thermometry study were purchased from Mircodiamant (Pureon), treated as reported in [1] (see Methods) and subsequently covalently functionalised in a stepwise manner with a L-tyrosyl-L-leucine dipeptide based on a recently reported method for the stable and biocompatible dipeptide functionalised detonation ND particles [2]. As a model system with highly comparable properties, and sufficient availability of the starting material, a moderately fluorescent HPHT NDs purchased from Microdiamant (Pureon) with comparable particle size and morphology, production and cleaning method, was employed as a model system to monitor the reaction progress and confirm dipeptide attachment by analytical methods. The only difference with the highly fluorescent HPHT NDs is the irradiation dose.

Due to a different initial surface termination of the fluorescent NDs starting material compared to the detonation nanodiamond (DND) particles used in the previously reported procedure [2], the synthetic procedure had to be adjusted and further optimized for working with small quantities of fluorescent HPHT ND. Firstly, carboxylic acid surface groups of ND were reduced with borane-tetrahydrofuran yielding ND-OH with an increased number of hydroxyl groups. These hydroxyl groups were subsequently used to anchor a short aromatic linker molecule to the particle surface using diazonium coupling technology as reported earlier [3]. Finally, alkyne-modified tyrosyl-leucine dipeptide was covalently bound to azide functionalised particles ND-O-N_3 by Cu(I)-catalysed "click" reaction giving dipeptide functionalised ND-O-Tyr-Leu (Figure 2a).

S.1.2 Methods for nanodiamond characterisation

FT-IR spectra were recorded with a Thermo Fischer Nicolet iS5 equipped with a unit for diffuse reflectance infrared fourier transform spectroscopy (DRIFTS). Particle size determination and zeta potential measurements performed in doubly distilled water (pH 6 – 7) using a Malvern Zetasizer Nano ZS (dynamic light scattering, backscattering mode) equipped with autotitrator MPT-2. The zeta potential was measured at the intrinsic pH of the sample. Particle size distributions were obtained as volume distribution (Dv(10), Dv(50), Dv(90)) using the Marquardt method. For centrifugation of the particles a Thermo Scientific Sorvall MTX 150 ultracentrifuge with a swing-out rotor was used.

S.1.3 Characterization of the fluorescent nanodiamond staring material model system

FT-IT(DRIFTS): $\widetilde{\nu}$ = 3550 (br, ν(O-H)), 1791 (s, ν(C=O)), 1629 (m), 1458 (m), 1383 (s, δ(O-H)), 1187 (w), 1089 (w, ν(C-O)), 714 (w), 672 (w), 649 (w), 505 (m) cm^{-1}. Zeta potential: −33.7 ± 1.10 mV (doubly distilled water, intrinsic pH = 5.2). Particle size (DLS): 10 % 76.1 ± 8.71 nm, 50 % 140 ± 14.6 nm, 90 % 247 ± 11.5 nm (doubly distilled water).

S.1.4 Reduction of the carboxylated starting material

NDs were isolated by ultracentrifugation (52 k rpm, 30 min) and washed twice with tetrahydrofuran before dispersing them in 10 mL of dry tetrahydrofuran under nitrogen atmosphere with the help of an ultrasonic bath. After 15 min of sonication, 2 mL of borane-tetrahydrofuran (1 M) were added dropwise. The dispersion was heated under reflux for 4 d. Then, hydrochloric acid (2 M) was added to the dispersion carefully until hydrogen generation stopped. The particles were subsequently centrifuged (16 k rpm, 15 min) and washed with acetone. After removing the supernatant, the particles were stirred in 20 mL of doubly distilled water for 2 h followed by two washing cycles with doubly distilled water to obtain **ND-OH**.

FT-IR (DRIFTS): $\widetilde{\nu}$ = 3560 (br, ν(O-H)), 2966 (m, ν(C-H)), 2903 (w, ν(C-H)), 1774 (m, ν(C=O)), 1632 (m), 1454 (w), 1375 (s, δ(O-H)), 1260 (s), 1095 (s, ν(C-O)), 1026 (m), 867 (w), 804 (s, γ(C-H)), 685 (w), 505 (w) cm^{-1}. Zeta potential: −25.8 ± 0.75 mV (doubly distilled water, intrinsic pH = 7.4). Particle size (DLS): 10 % 76.7 ± 7.49 nm, 50 % 146 ± 17.3 nm, 90 % 265 ± 15.9 nm (doubly distilled water).

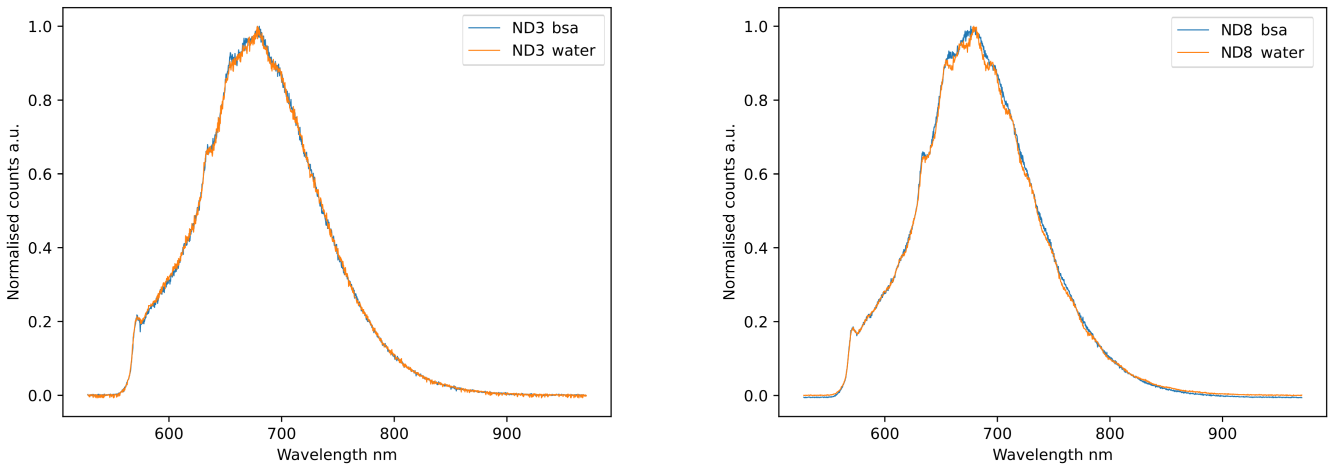

(a) (b)

Figure S. 1: Fluorescence spectra of NDs in water and in BSA. 60 seconds, 2 µW 513 nm excitation, background corrected

S.1.5 Attachment of the aromatic azide linker

ND-OH was dispersed in 30 mL of doubly distilled water using an ultrasonic bath for 30 min. Afterwards, the dispersion was heated up to 80 °C and 50 mg of 4-(2-azidoethyl)phenylamine (0.31 mmol) and 50 µL of amyl nitrite (45.7 mg, 0.39 mmol) were added. The dispersion was stirred overnight at 80 °C and then allowed to cool to room temperature. Followed by isolation using ultracentrifugation (52 k rpm), the particles were extensively washed with acetone (3x), dimethylformamide (3x), acetone (3x), dimethylformamide (3x) and finally doubly distilled water (3x) yielding azide functionalised **ND-O-N$_3$**.

FT-IR (DRIFTS): $\widetilde{\nu}$ = 3445 (br, ν(O-H)), 2961 (m, ν(C-H)), 2927 (w, ν(C-H)), 2864 (w, ν(C-H)), 2102 (m, ν(N$_3$)), 1762 (m, ν(C=O)), 1633 (m), 1452 (w), 1373 (s, δ(O-H)), 1261 (s), 1101 (s, ν(C-O)), 1055 (m), 863 (w), 807 (s, δ(C-H$_{1,4\text{-arom}}$)), 706 (w), 685 (w), 530 (w) 507 (w) cm^{-1}. Zeta potential: −20.2 ± 0.23 mV (doubly distilled water, intrinsic pH = 6.7). Particle size (DLS): 10 % 92.7 ± 1.52 nm, 50 % 154 ± 3.51 nm, 90 % 243 ± 8.54 nm (doubly distilled water).

S.1.6 Conjugation with L-Tyr-L-Leu dipeptide by 'click' reaction

ND-O-N$_3$ was washed twice with dimethylformamide (52 k rpm, 30 min) before redispersing it in 10 mL of degassed dimethylformamide. The dispersion was degassed under nitrogen atmosphere by bubbling nitrogen through it and simultaneously sonicating for 15 min. Then, 13.3 mg of copper (II) sulfate (0.08 mmol) were added and the degassing procedure was repeated. Next, 33.3 mg of sodium ascorbate (0.17 mmol) were added and the dispersion was stirred for 1 h before addition of 20.0 mg of (S)-2-Amino-3-(4-(prop-2-yn-1-yloxy)phenyl)propanoyl-L-leucinate **Tyr(*O*-propargyl)-Leu** (0.06 mmol). The dispersion was stirred under nitrogen atmosphere and at room temperature for 5 d. Afterwards, the particles were ultracentrifuged (52 k rpm, 30 min) and washed consecutively with dimethylformamide (3x), acetone (3x), ethylenediaminetetraacetic acid (EDTA, 0.1 M) solution (3x) and doubly distilled water (3x) to yield **ND-O-Tyr-Leu**.

FT-IR (DRIFTS): $\widetilde{\nu}$ = 3444 (br, ν(O-H)), 2962 (m, ν(C-H)), 2876 (w, ν(C-H)), 1643 (m, ν(C=O), amide I), 1509 (w, ν(C=C$_{\text{arom}}$)), 1458 (w), 1374 (s, δ(O-H)), 1263 (m), 1217 (w, ν(C-O)), 1179 (w), 1098 (m, ν(C-O)), 942 (w), 811 (m, δ(C-H$_{1,4\text{-arom}}$)), 711 (w), 685 (w), 529 (w) cm^{-1}. Zeta potential: −21.0 ± 0.25 mV (doubly distilled water, intrinsic pH = 6.5). Particle size (DLS): 10 % 92.6 ± 6.61 nm, 50 % 154 ± 3.00 nm, 90 % 244 ± 10.6 nm (doubly distilled water).

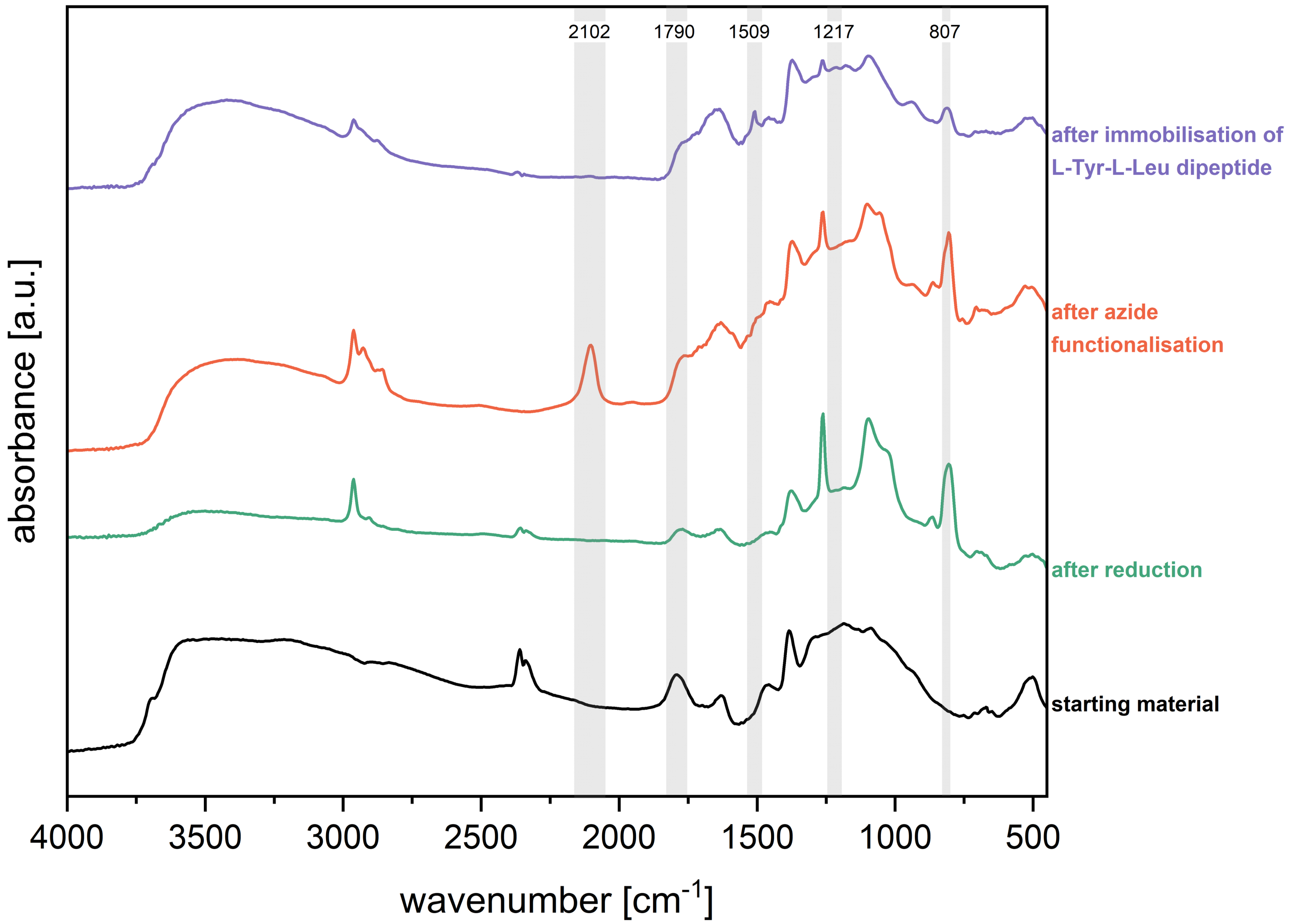

Figure S. 2: DRIFT spectra of the different surface functionalisation stages of fluorescent nanodiamond (model system). Gray areas highlight significant bands.

S.2 Derivation surface potential sensing

Electrical field inside the nanodiamond

Despite the diversity of ND shapes, we will assume first that they are all spheres to see if such a simple approach is enough to describe the effect of electrical field on the ND ODMR spectra. Then, due to spherical symmetry, the potential outside the charged ND is written as follows:

$$\psi(q,r) = \frac{1}{4\pi\varepsilon_{rH_2O}\varepsilon_0} \times \frac{q}{r}. \quad \text{(E.1)}$$

With ε_{rH_2O} being the relative permittivity of water and ε_0 the permittivity of free space respectively, r, the distance from the centre.

In the case of the surface potential (ψ_0) of a particle of charge Q, with R being the radius of the particle, we have:

$$\psi_0 = \frac{1}{4\pi\varepsilon_{rH_2O}\varepsilon_0} \times \frac{Q}{R}. \quad \text{(E.2)}$$

Experimental studies using NV inclusions in ND show that the electric field inside ND is non-zero. Since the causes of charges in ND are processes occurring at the interface "ND - liquid", as a first approximation we use the assumption that the charge density inside ND, $\rho(r)$, is distributed linearly and has a maximum value on its surface:

$$\rho(r) = \rho_{max}\frac{r}{R}. \quad \text{(E.3)}$$

We find ρ_{max} from the normalisation condition:

$$Q = \int_V \rho(r)dV = \pi\rho_{max}R^3. \quad \text{(E.4)}$$

To find the field distribution inside the ND, we apply Gauss theorem,

$$\int_V \rho(r)dV = \oint D_n dS. \quad \text{(E.5)}$$

Where S is a spherical surface with a centre coinciding with the centre of the ND and radius r. $D_n = \varepsilon_{rH20}\varepsilon_{rd}E_n$ is the normal component of the electrical induction vector to the surface S. E_n is the normal component of electrical field strength to the spherical surface S. ε_{rd} is the relative permittivity of diamond. Due to the spherical symmetry of the system and the negative polarity of the stern layer on ND surface, the directions of the vectors D, E and r coincide, and their modules are equal to the components normal to the S surfaces.

Furthermore, we have

$$\oint D_n dS = 4\pi r^2 \varepsilon_0 \varepsilon_{rd} E(r). \tag{E.6}$$

Using equation E.5 we obtain

$$E(r) = \frac{\varepsilon_{rH_2O}\psi_0}{\varepsilon_{rd}R^3} r^2. \tag{E.7}$$

For our ND we use $\varepsilon_{rH_2O} = 81$, $\varepsilon_{rd} = 5.7$ [4, 5, 6] and $R = 50 \times 10^{-9}m$. For the case of ND immersed into a 300 g/L BSA solution, we can calculate the permittivity by Wagner's equation given by $\phi(e_q - e_w)/(e_q + 2e_b) = (e_{ap} - e_w)/(e_a p + 2e_w)$, where e_q, e_w, and e_{ap} are dielectric permittivity of protein, water, and the protein solution, respectively, and phi is the volume fraction of protein occupied in the solution. Using the values of $e_q = 2$, $e_w = 81$, and $\phi = 0.3$, the relative dielectric permittivity of the protein solution is approximately 52.

Electrical field sensing with NVs

If we neglect the random magnetic field and the hyperfine interaction, the Hamiltonian is [6, 7, 8] :

$$H = (D_{gs} + \Pi_z)S_z^2 + \Pi_x(S_y^2 - S_x^2) + \Pi_y(S_xS_y + S_yS_x). \tag{E.8}$$

Here, the vector z is the NV axis, the vector x is defined such that one of the carbon-vacancy bonds lies in the x-z plane, S is the electronic spin-1 operator of the NV. $\Pi_{\{x,y\}} = d_\perp E_{\{x,y\}}$ and $\Pi_z = d_{\|} E_z$ and $\{d_{\|}, d_\perp\} = \{0.35; 17\}\, Hz\, cm/V$ with E being the electrical field [9].

And the energy levels are

$$D_{gs} - \sqrt{\Pi_x^2 + \Pi_y^2} + \Pi_z, D_{gs} + \sqrt{\Pi_x^2 + \Pi_y^2} + \Pi_z. \tag{E.9}$$

With the splitting, $s = 2\sqrt{\Pi_x^2 + \Pi_y^2}$, using $E_\perp = \sqrt{E_x^2 + E_y^2}$ we have

$$s = 2d_\perp E_\perp. \tag{E.10}$$

Modelling the ND spectra from a 100 NVs

1. Sphere of 100 nm diameter divided into shells of 5 nm

2. Decreasing surface electrical fields are $\overrightarrow{E_s} = \{\overrightarrow{E_{s1}}, \overrightarrow{E_{s2}}, ...\}$ created using equation E.7.

   (a) The ODMR spectra from a 25 nm ND with a very shallow NV exposed the surface (see figure S. 3), suggests that the $E_\perp$ could go up to $1.22 \times 10^6 V/cm$. The maximum value of $2 \times 10^6 V/cm$ (corresponding to a surface potential of 0.7 V) for the magnitude of the surface electrical ($\|\overrightarrow{E_s}\|$) field was chosen as higher values did not significantly improved the fitting (*i.e.* Rsquare). Indeed greater values lead to splitting greater than the width of the ODMR spectra.

   (b) We also assume that most charges on the ND's surface are negative as as the particles showed negative zeta potential.

   (c) We assume that the interaction with the proteins does not change the sign of the potential.

3. For a first shell, the most shallow one, the sum of the surface and bulk electrical field are calculated:

$$\overrightarrow{E_{sh1}} = \overrightarrow{E_{s1}} + \overrightarrow{E_b} \tag{E.11}$$

   (a) We assume the bulk electrical field is negative as modelled by Dinani *et al.* [10] but further investigation are needed to confirm that it is the case for our diamond as the laser light can ionise the nitrogen atoms N$^-$ into N$^+$.

   (b) We kept the value of the magnitude of the bulk electrical field $\|\overrightarrow{E_b}\|$ constant.

      i. This is because unlike the FWHM that increases by 24% with BSA (bovine serum albumin 300g/L, n= 7), the splitting only fluctuate by approximately 13% (1.5 MHz standard deviation for a 12 MHz average fitting, N= 13) among the different NDs and conditions (*i.e.* in water and BSA see figure S. 4). If the bulk field was increasing because of the increase of charge density in the ND (from water to BSA), the splitting would increase

as shown by Yu *et al.* [6]. Further, in figure S. 3, the core NV signal splitting only fluctuates by 11% between BSA and water, while the surface NV increases its splitting by 39%.

(c) We fixed the $\|\overrightarrow{E_b}\|$ to $2.45 \times 10^5 V/cm$ as it is the average magnitude across 7 different 100 nm NDs in water estimated by the model when $\|\overrightarrow{E_b}\|$ was set as a free parameter.

i. It is worth noting that the other phenomenon might be the cause of the splitting (*i.e.* strain) but we assume that such phenomenon will not change upon a change in surface chemistry as the splitting does not significantly change across the different NDs and conditions (in water and in BSA).

4. $\overrightarrow{E_{sh1}}$ is applied to a NV and the ODMR spectra is calculated according to equation E.9. The polar and the azimuthal angles determines the alignment of the $\overrightarrow{E_{sh1}}$ with the NV z axis.

5. The next NV in the shell is rotated into one of the 4 possible orientations of the NV and $\overrightarrow{E_{sh1}}$ is applied to get the next ODMR NV's spectra with the z axis being aligned with the NV z axis as $\overrightarrow{E_{s1}}$ is normal to the surface.

6. Once all the NVs in the shell have been addressed, the ODMR spectra of the entire shell is calculated from the sum of the NVs spectra within that shell.

7. The same is repeated with a decreasing surface electrical field $\overrightarrow{E_{s2}}$ according to equation E.7 for a 5 nm deeper shell until there are no NVs left

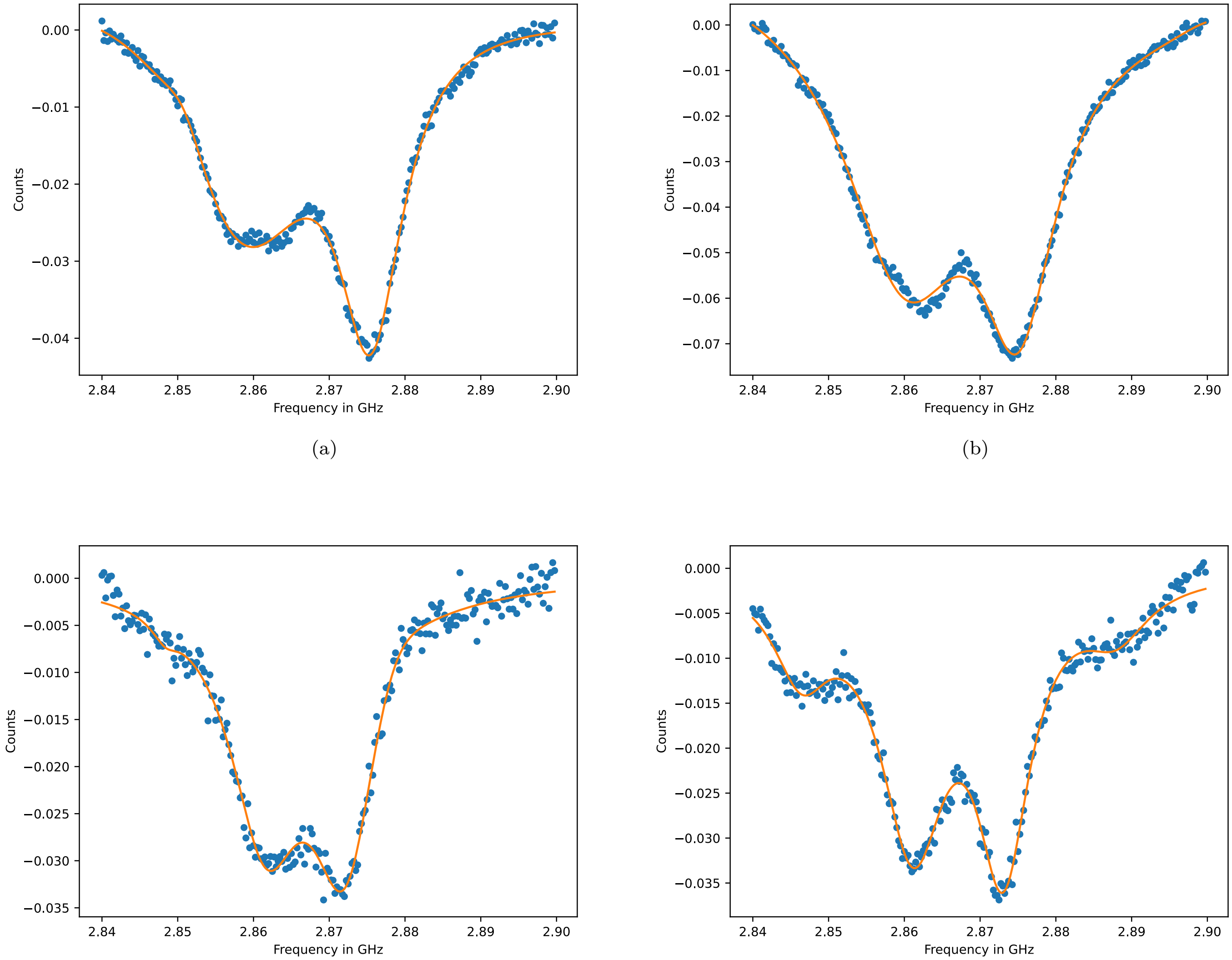

(a) (b) (c) (d)

Figure S. 3: ODMR spectra of 25 nm NDs at 37°C in air (a) and water (b) and BSA (c, d) solution with a 4 Lorentzian dip fit. The fact that the larger splitting is disappearing (c) and reappears 7 min later (d) suggest that the NV is close to the surface as unstable NVs are typically close to the surface [11, 12] (also the counts increase by 15% at T= +7 min). The shallow NV shows an greater splitting (39% increase) when going from water to BSA. Using equation E.10 $E_\perp = 1.22 \times 10^6$ V/cm (the field could be stronger in the E_z component but the uncertainty of the measurement prevents us to have an estimation). The smaller splitting corresponding to the NV closer to the centre changes by only 11% (1.3 MHz).

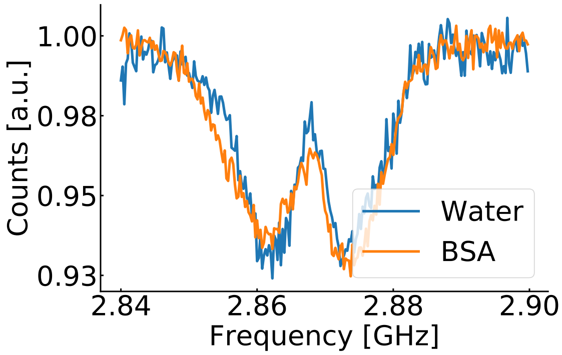

(a)

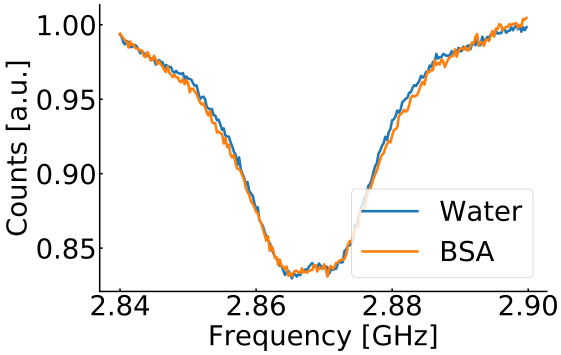

(b)

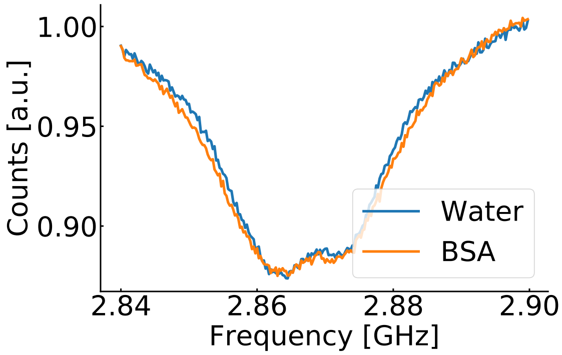

(c)

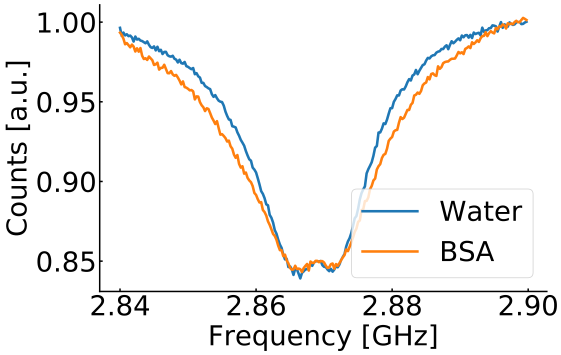

(d)

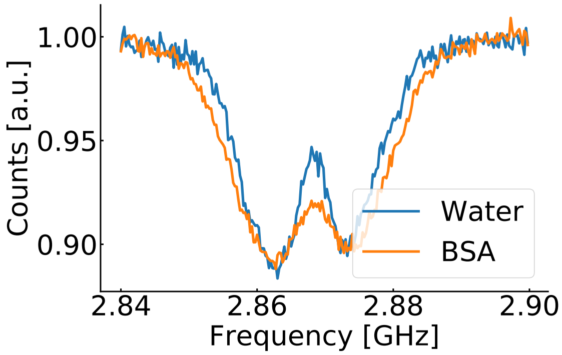

(e)

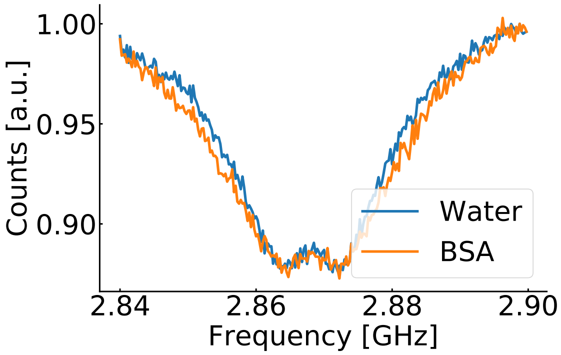

(f)

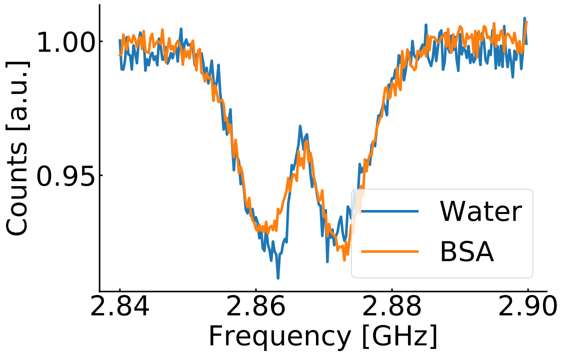

(g)

Figure S. 4: Examples of ODMR spectra from the same set of unfunctionalised 100 nm HPHT NDs at 37°C in water and BSA solution (normalised to the same contrast)

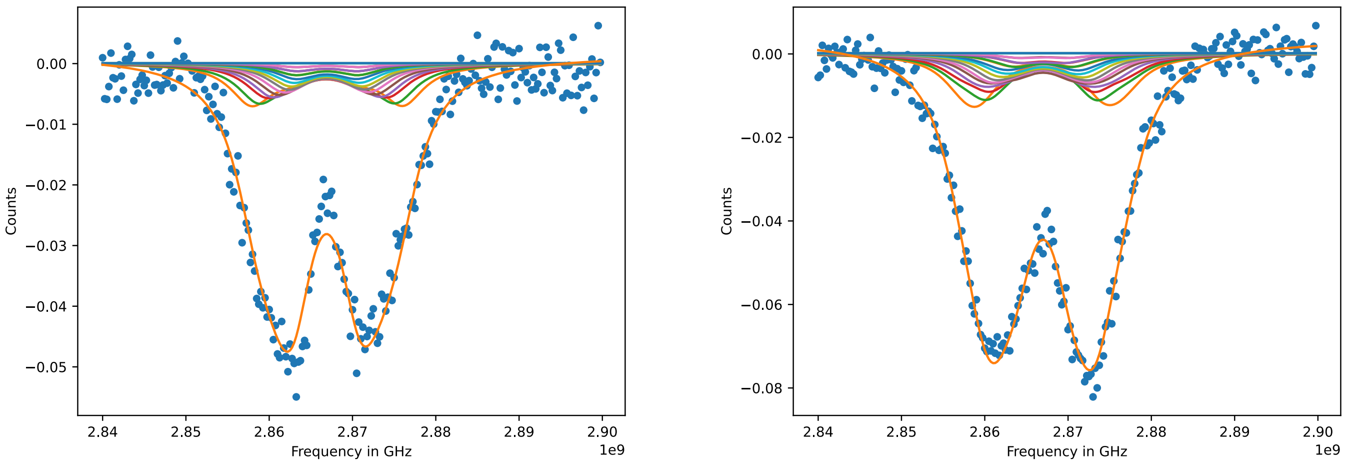

(a) (b)

Figure S. 5: ODMR spectra of the same 100 nm HPHT ND at 37°C in water (a) and BSA (b) solutions. Spectra fitted with the model.

Modelling output

The surface potential obtained is on average -280 mV (corresponding to a $\|\overrightarrow{E_s}\|$of $8 \times 10^5 V/cm$), which is about 3.5 times larger than the zeta potential measured of these particles measured in water pH 5 (-80 mV). As shown in figure S. 5, the surface potential typically increases when the ND is exposed to BSA (14% increase in average, n= 7). The estimations of the surface potential in water is in the same order of magnitude as contact potential measurements on oxygen terminated NDs using Kelvin Probe Force Microscopy (KPFM) [13] also, as expected, the zeta potential of these NDs in water is lower than the surface potential estimations (-80 mV) [14, 15].

S.3 Details on the Thermal Echo (TE) Scheme

We consider a three-level system (see Figure S. 6a) whose dynamics in the basis $\{|+1\rangle, |0\rangle, |-1\rangle\}$ is governed by the Hamiltonian

$$\begin{aligned} H_0 &= (D+\delta)S_z^2 + \Delta S_z + 2\Omega \cos(\omega_c t + \phi) S_x \\ &= \begin{bmatrix} (D+\delta)+\Delta & \Omega\sqrt{2}\cos(\omega_c t+\phi) & 0 \\ \Omega\sqrt{2}\cos(\omega_c t+\phi) & 0 & \Omega\sqrt{2}\cos(\omega_c t+\phi) \\ 0 & \Omega\sqrt{2}\cos(\omega_c t+\phi) & (D+\delta)-\Delta \end{bmatrix}, \end{aligned} \tag{E.12}$$

where $S_i, i = x, y, z$ are the spin 1 operators, D is the zero-field splitting at the temperature we expect, δ is a change in D due to a slightly different actual temperature that we want to measure with $\delta \approx -(2\pi)74KHz/K$ [16, 17], Δ is the splitting between the $m_s = \pm 1$ states in the presence of a magnetic field and/or a detuning, e.g., due to a hyperfine interaction between the electron spin of the NV center and the nuclear spin of the nitrogen. It is typically small, i.e., $\Omega > \Delta$ in our experiments. We apply a control field with a Rabi frequency 2Ω, driving angular frequency ω_c and a phase ϕ.

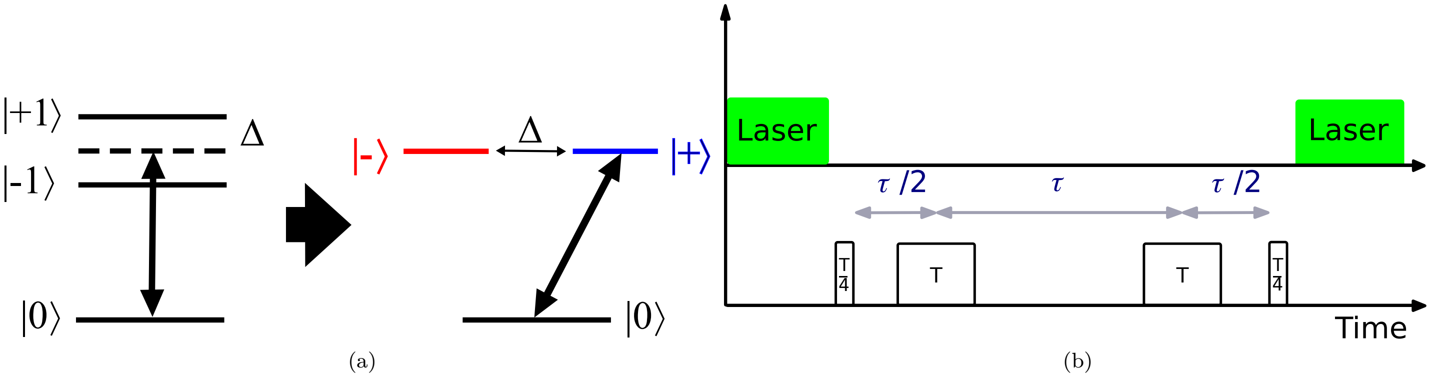

Figure S. 6: **(a)** Level scheme for low-field nano-NMR sensing, which we consider in this work and the corresponding scheme in the dressed basis with $|\pm\rangle = (|+1\rangle \pm |-1\rangle)/\sqrt{2}$. The state $|-\rangle$ is decoupled if states $|+1\rangle$ and $|-1\rangle$ are degenerate, i.e., $\Delta = 0$. **(b)** Thermal echo scheme. The system is inilitialised to state $|0\rangle$ by a laser pulse. We create a superposition between $|0\rangle$ and $|+\rangle$ with a microwave $T/4$ pulse (taking $T = \pi/\overline{\Omega} \approx \pi/\Omega$). Applying pulses of duration T takes $|+\rangle \rightarrow -|+\rangle$ and back, thus refocusing the effect of Δ. During the free evolution, the coherence accumulates a phase $\delta t, t = n\tau$, where n is the number of applied pulses, which depends on a frequency shift δ from the expected zero-field splitting D, e.g., due to temperature change. Finally, the coherence is mapped back to the populations by a $T/4$ pulse, allowing the detection of δ and temperature by state-dependent fluorescence when applying a laser pulse.

We move to the interaction basis, defined by $H_0^{(1)} = \omega_c S_z^2$

$$\begin{aligned} H_1 &= U_0^{(1)}(t)^\dagger H_0 U_0^{(1)}(t) - iU_0^{(1)}(t)^\dagger \partial_t U_0^{(1)}(t) \\ &= \begin{bmatrix} (D+\delta-\omega_c)+\Delta & \Omega\sqrt{2}\cos{(\omega_c t+\phi)}e^{i\omega_c t} & 0 \\ \Omega\sqrt{2}\cos{(\omega_c t+\phi)}e^{-i\omega_c t} & 0 & \Omega\sqrt{2}\cos{(\omega_c t+\phi)}e^{-i\omega_c t} \\ 0 & \Omega\sqrt{2}\cos{(\omega_c t+\phi)}e^{i\omega_c t} & (D+\delta-\omega_c)-\Delta \end{bmatrix} \\ &\approx \begin{bmatrix} \delta+\Delta & \frac{\Omega}{\sqrt{2}}e^{-i\phi} & 0 \\ \frac{\Omega}{\sqrt{2}}e^{i\phi} & 0 & \frac{\Omega}{\sqrt{2}}e^{i\phi} \\ 0 & \frac{\Omega}{\sqrt{2}}e^{-i\phi} & \delta-\Delta \end{bmatrix} \end{aligned} \tag{E.13}$$

where $U_0^{(1)}(t) = \exp\left(-iH_0^{(1)}t\right) = \exp{(-i\omega_c t S_z^2)}$, we assumed that resonance $\omega_c = D$ and applied the rotating-wave approximation (RWA), neglecting the terms rotating at $2\omega_c t$ in the last row.

It is useful to consider the evolution of the system in the dressed basis, where the basis states are given by $|+\rangle = (|+1\rangle + |-1\rangle)/\sqrt{2}$, $|0\rangle$, and $|-\rangle = (|+1\rangle - |-1\rangle)/\sqrt{2}$. The respective Hamiltonian, describing the time evolution of the system in the basis $\{|+\rangle, |0\rangle, |-\rangle\}$, is given by

$$H_{\mathrm{dr}} = \begin{bmatrix} \delta & \Omega e^{-i\phi} & \Delta \\ \Omega e^{i\phi} & 0 & 0 \\ \Delta & 0 & \delta \end{bmatrix}. \tag{E.14}$$

During the free evolution, the system in the dressed basis is characterized by the Hamiltonian

$$H_{\mathrm{free}} = \begin{bmatrix} \delta & 0 & \Delta \\ 0 & 0 & 0 \\ \Delta & 0 & \delta \end{bmatrix}. \tag{E.15}$$

The dynamics of the system then for time t is then described by the propagator

$$\begin{aligned} U_{\mathrm{free}}(t) &= \exp{(-iH_{\mathrm{free}}t)} \\ &= \begin{bmatrix} e^{-i\delta t}\cos(\Delta t) & 0 & -ie^{-i\delta t}\sin(\Delta t) \\ 0 & 1 & 0 \\ -ie^{-i\delta t}\sin(\Delta t) & 0 & e^{-i\delta t}\cos(\Delta t) \end{bmatrix}. \end{aligned} \tag{E.16}$$

In the following, we describe the system evolution during the pulses. We assume for simplicity that the pulses we apply are short, so we can neglect the effect of Δ and δ during them ($\Omega \gg \Delta, \delta$). Then, the Hamiltonian during a pulse can be approximated by

$$H_{\mathrm{pulse}} \approx \begin{bmatrix} 0 & \Omega e^{-i\phi} & 0 \\ \Omega e^{i\phi} & 0 & 0 \\ 0 & 0 & 0 \end{bmatrix}, \tag{E.17}$$

where we neglected the effect of $\Delta, \delta \ll \Omega$ during the pulse. In the case when Δ is comparable to the Rabi frequency Ω, one can use the phase ϕ as a control parameter to apply robust low-field sequences for dynamical decoupling (LDD), as shown in [18]. The evolution due to a pulse with duration t on the system is described by a propagator

$$\begin{aligned} U_{\mathrm{pulse}}(t) &= \exp{(-iH_{\mathrm{pulse}}t)} \\ &= \begin{bmatrix} \cos(\Omega t) & -i\sin(\Omega t) & 0 \\ -i\sin(\Omega t) & \cos(\Omega t) & 0 \\ 0 & 0 & 1 \end{bmatrix}, \end{aligned} \tag{E.18}$$

where we assumed for simplicity of presentation and without loss of generality $\phi = 0$. In the following, we describe how the thermal echo (TE) sequence works.

First, the system is initialized in state $|0\rangle$ by optical pumping with a green laser pulse (see the main text) with the initial density matrix given by

$$\rho_0 = \begin{bmatrix} 0 & 0 & 0 \\ 0 & 1 & 0 \\ 0 & 0 & 0 \end{bmatrix}. \tag{E.19}$$

Then, we apply a preparation pulse with a duration $T/4$, where $T = \pi/\overline{\Omega} \approx \pi/\Omega$, where $\overline{\Omega} = \sqrt{\Omega^2 + \Delta^2}$ and the approximation is valid due to the assumption that $\Delta \ll \Omega$. The preparation pulse creates a maximum coherent superposition between states $|0\rangle$ and $|+\rangle$, which is sensitive to the detunings δ and Δ and is useful for sensing. The propagator of the

preparation pulse with duration $t = T/4$ and the system density matrix after it are given by

$$U_{\text{prep}} = U_{\text{pulse}}(T/4) = \begin{bmatrix} \frac{1}{\sqrt{2}} & -\frac{i}{\sqrt{2}} & 0 \\ -\frac{i}{\sqrt{2}} & \frac{1}{\sqrt{2}} & 0 \\ 0 & 0 & 1 \end{bmatrix}, \quad \text{(E.20)}$$

$$\rho_{\text{prep}} = U_{\text{prep}}\rho_0 U_{\text{prep}}^{\dagger} = \begin{bmatrix} \frac{1}{2} & -\frac{i}{2} & 0 \\ \frac{i}{2} & \frac{1}{2} & 0 \\ 0 & 0 & 0 \end{bmatrix}. \quad \text{(E.21)}$$

The preparation $T/4$ pulse is followed by free evolution periods, characterised by a pulse spacing time τ (see Figure S. 6) and pulses with duration T. The latter refocus the effect of the detuning Δ, increasing the coherence time and allowing for better sensitivity to δ and thus better temperature sensing.

It is useful to characterize the system evolution after the preparation pulse in the interaction frame of the pulses. The Hamiltonians in the free evolution periods (1)-(3) are given by:

Period (1): before the first refocusing T pulse:

$$H_{\text{int},1} = H_{\text{free}} = \begin{bmatrix} \delta & 0 & \Delta \\ 0 & 0 & 0 \\ \Delta & 0 & \delta \end{bmatrix}, \qquad t_1 \approx \frac{\tau}{2}. \quad \text{(E.22)}$$

Period (2): after the first refocusing T pulse and before the second refocusing T pulse:

$$H_{\text{int},2} = U_{\text{int},2} H_{\text{free}} U_{\text{int},2}^{\dagger} = \begin{bmatrix} \delta & 0 & -\Delta \\ 0 & 0 & 0 \\ -\Delta & 0 & \delta \end{bmatrix}, \quad \text{(E.23)}$$

$$U_{\text{int},2} = U_{\text{pulse}}(T) = \begin{bmatrix} -1 & 0 & 0 \\ 0 & -1 & 0 \\ 0 & 0 & 1 \end{bmatrix}, \qquad t_2 \approx \tau.$$

Period (3): after the second refocusing T pulse:

$$H_{\text{int},3} = U_{\text{int},3} H_{\text{free}} U_{\text{int},3}^{\dagger} = \begin{bmatrix} \delta & 0 & \Delta \\ 0 & 0 & 0 \\ \Delta & 0 & \delta \end{bmatrix}, \quad \text{(E.24)}$$

$$U_{\text{int},3} = U_{\text{pulse}}(T) U_{\text{pulse}}(T) = \begin{bmatrix} 1 & 0 & 0 \\ 0 & 1 & 0 \\ 0 & 0 & 1 \end{bmatrix}, \ t_3 \approx \tau/2.$$

It is evident that the effect of Δ during periods (1) and (3) is compensated during period (2) when the respective signs of the Hamiltonian elements that contain Δ are opposite. The total evolution can be found by finding the average Hamiltonian of all individual interaction Hamiltonians as they commute, $[H_{int,i}, H_{int,j}] = 0$. We thus obtain

$$\overline{H}_{\text{int}} = \frac{\sum_p H_{int,p} t_p}{\sum_p t_p} = \begin{bmatrix} \delta & 0 & 0 \\ 0 & 0 & 0 \\ 0 & 0 & \delta \end{bmatrix}, \quad \text{(E.25)}$$

$$U_{\text{sense}}(t) = \exp\left(-i\overline{H}_{\text{int}} t\right) = \begin{bmatrix} e^{-i\delta t} & 0 & 0 \\ 0 & 1 & 0 \\ 0 & 0 & e^{-i\delta t} \end{bmatrix}, \ t = n\tau,$$

where $U_{\text{sense}}(t)$ is the propagator that describes the evolution of the system for a total sensing time of $t = n\tau$, where n is the number of applied refocusing pulses ($n = 2$ in our particular example). We have shown that $U_{\text{int},3}$ is the identity operator, so the interaction basis of the pulses at the end of the process is the same as the basis $\{|+\rangle, |0\rangle, |-\rangle\}$. This is also true after every second T pulse if we apply more pulses. Thus, the propagator $U_{\text{sense}}(t)$ can also be used to describe the time evolution in the basis $\{|+\rangle, |0\rangle, |-\rangle\}$. We confirm this by calculating directly the propagator in the basis $\{|+\rangle, |0\rangle, |-\rangle\}$ for a free evolution period $\tau/2$, followed by a pulse with duration T, free evolution period τ, a second T pulse, and a final free evolution period $\tau/2$. It is straightforward to show that $U_{\text{sense}}(t) = U_{\text{free}}(\tau/2) U_{\text{pulse}}(T) U_{\text{free}}(\tau) U_{\text{pulse}}(T) U_{\text{free}}(\tau/2)$.

The density matrix after the sensing time is then given by

$$\rho_{\text{sense}}(t) = U_{\text{sense}}(t)\rho_{\text{prep}} U_{\text{sense}}^{\dagger}(t) \quad \text{(E.26)}$$
$$= \begin{bmatrix} \frac{1}{2} & -\frac{i}{2}e^{-i\delta t} & 0 \\ \frac{i}{2}e^{i\delta t} & \frac{1}{2} & 0 \\ 0 & 0 & 0 \end{bmatrix}, \ t = n\tau.$$

In a last step, we apply a readout $T/4$, which maps the accumulated phase back onto the populations of states $|0\rangle$ and $|+\rangle$. The density matrix afterwards takes the form

$$\rho_{\text{read}}(t) = U_{\text{pulse}}(T/4)\rho_{\text{sense}}(t) U_{\text{pulse}}^{\dagger}(T/4) \quad \text{(E.27)}$$
$$= \begin{bmatrix} \cos^2\left(\frac{\delta t}{2}\right) & -\frac{1}{2}\sin(\delta t) & 0 \\ -\frac{1}{2}\sin(\delta t) & \sin^2\left(\frac{\delta t}{2}\right) & 0 \\ 0 & 0 & 0 \end{bmatrix}, \ t = n\tau.$$

The result above is obtained for a readout pulse with phase $\phi = 0$. The pulse phase can also be alternated to $\phi = \pi$, which exchanges the final populations of states $|0\rangle$ and $|+\rangle$. We infer the population of state $|0\rangle$ after the readout pulse by applying a laser pulse and detecting the resulting fluorescence (see main

text). The difference between the fluorescence signals from the second and first phase alternating measurements is then

$$S \sim \cos^2\left(\frac{\delta t}{2}\right) - \sin^2\left(\frac{\delta t}{2}\right) = \cos\left(\delta t\right). \qquad \text{(E.28)}$$

The signal oscillates when we vary the sensing time $t = n\tau$, which allows us to sense δ and thus small changes in temperature. The linewidth is $\sim 1/T_2$ with T_2 being the coherence time of the applied decoupling sequence, which can reach hundreds of μs [18] and is limited by the relaxation time T_1 of the NV centers with the latter being up to several ms.

S.4 Setup and other controls

Condition	Zeta potential [mV]
200 nM KCl	−32.8 ± 0.81
500 nM KCl	−34.4 ± 2.48
1 µM KCl	−33.5 ± 0.27
10 mM PBS[a] (pH = 7.4)	−26.2 ± 1.84
1% mPEG-OH[b] in H_2O	−16.8 ± 0.40
pH = 8.9	−28.0 ± 0.30
pH = 7.5	−23.1 ± 0.80
pH = 6.8	−26.7 ± 0.75
pH = 6.0	−29.9 ± 0.46
pH = 4.7	−29.7 ± 0.66
pH = 3.6	−33.6 ± 0.56

Table 1: Zeta potential measurement on the functionalised HPHT NDs (the model NDs that were available in greater quantity, see the functionalisation section for more details). [a] PBS: phosphate-buffered saline; [b] mPEG-OH: polyethylene glycol monomethyl ether, average molecular weight 350g/mol.

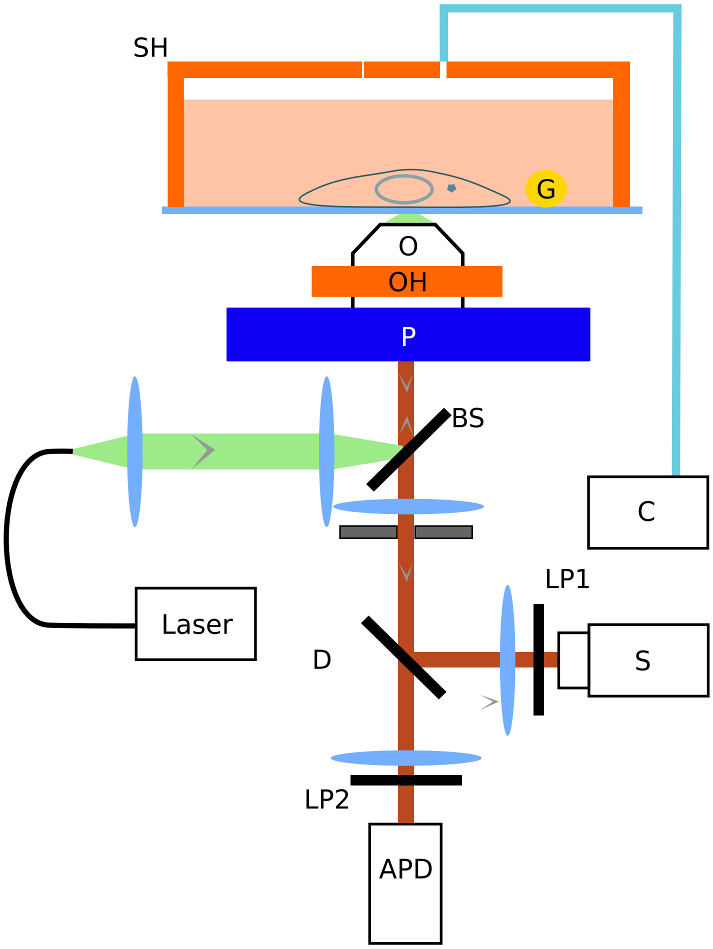

Figure S. 7: Simplified description of the setup. Laser: 513 nm, APD: avalanche photodiode, BS: beam sampler, C: CO2 controller, D: dichroic mirror, (T70%, R30%) G: gold wire, LP1: long pass (561 nm cut off), LP2: long pass (590 nm cut off), O: objective, OH: objective heater, P: XYZ piezo stage, S: spectrometer, SH: heated inset

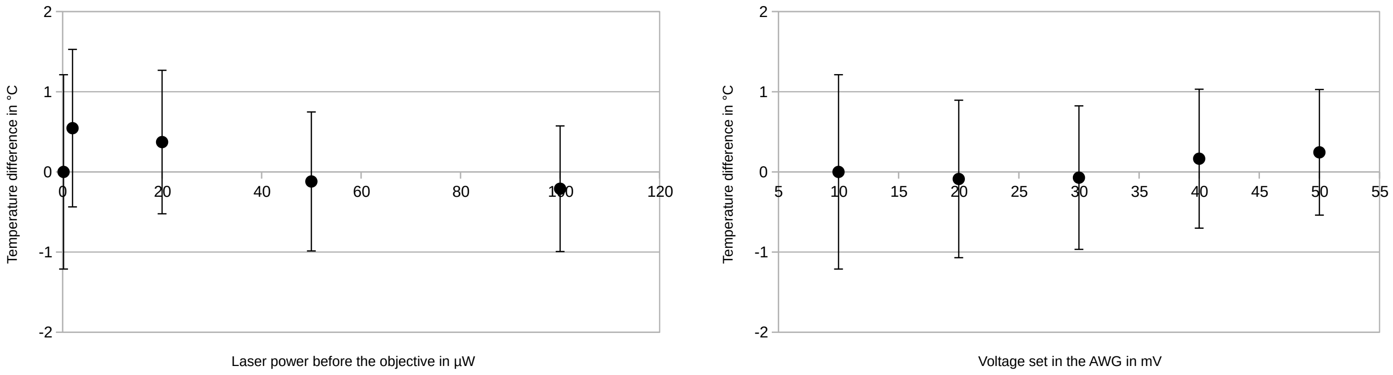

(a) (b)

Figure S. 8: Confirmation of the ND temperature stability independent of the laser or microwave power used (HPHT ND, CW-ODMR, see experimental section in main text for details)

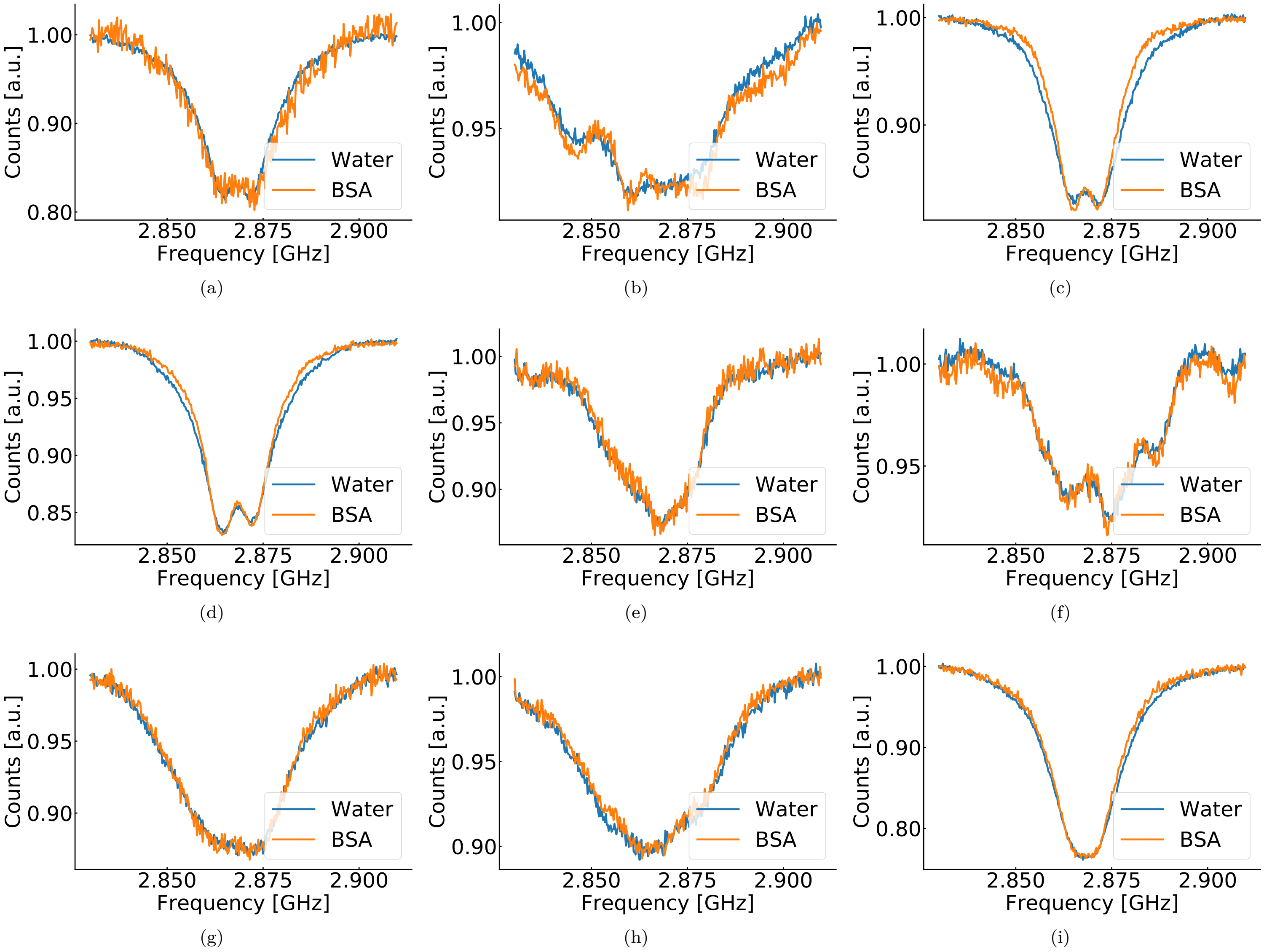

Figure S. 9: Examples of ODMR spectra of the same set of dipeptide functionalised 100 nm HPHT NDs at 37°C in water and BSA solution (normalised to the same contrast).

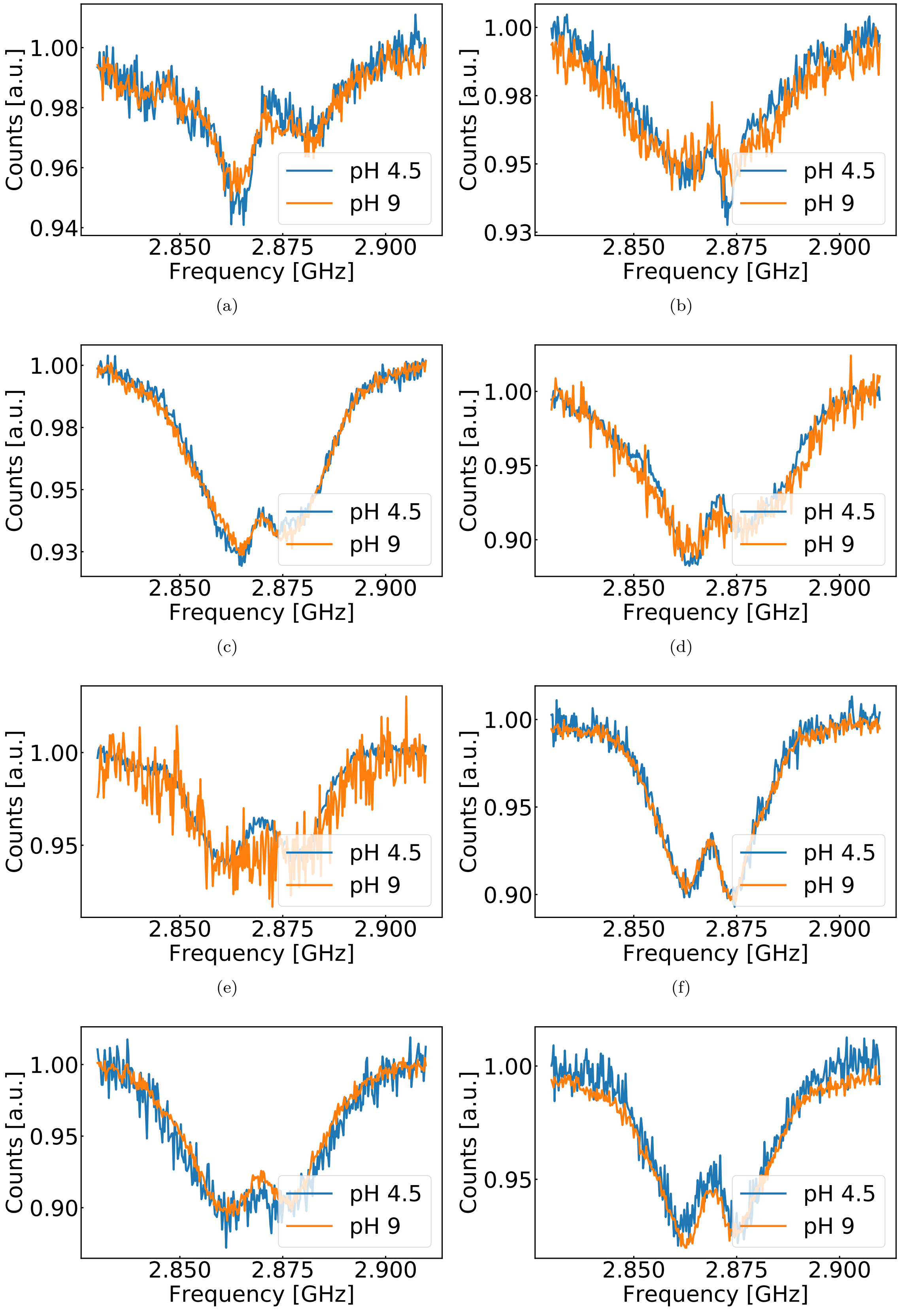

(a) (b) (c) (d) (e) (f) (g) (h)

Figure S. 10: Comparison of ODMR spectra from the same set of functionalised HPHT NDs in pH 4.5 and pH 9 at room temperature (normalised to the same contrast).

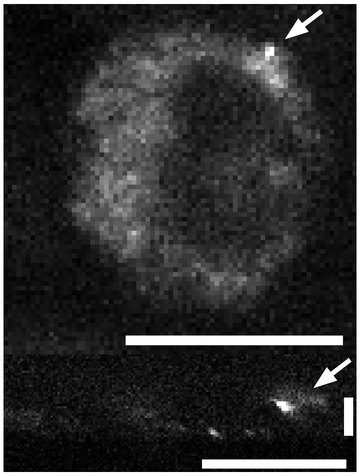

Figure S. 11: Confocal pictures of cells. NDs indicated with an arrow For all confocal images : 513 nm excitation, 2 µW before the objective, horizontal and vertical scale bars: 10 µm.

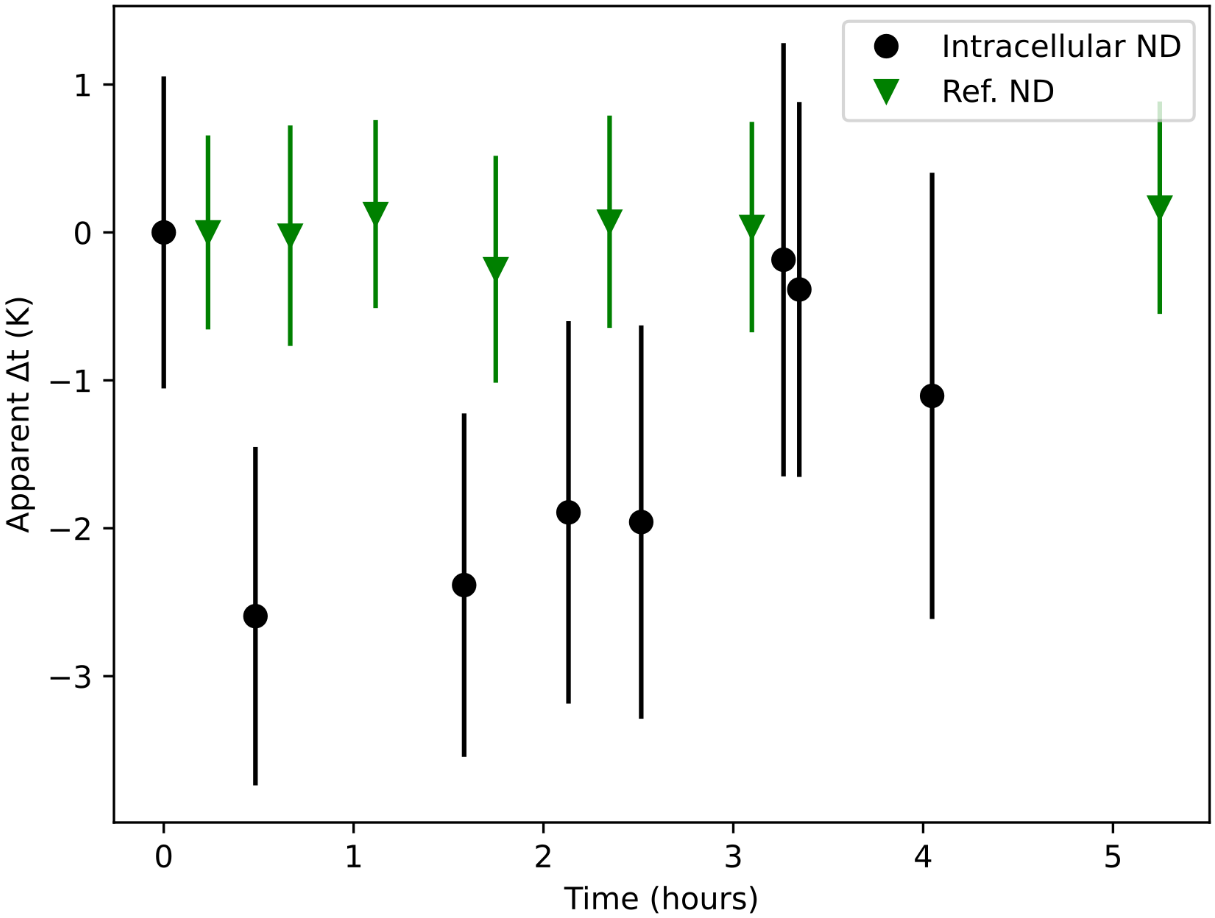

Figure S. 12: No artefact and stable temperature reading when the ND is outside the cell (Ref. ND). Intracellular nanothermometry with CW-ODMR inside a live macrophage (ND termed: intracellular ND, see experimental section in main text for details). Simultaneously, an ND immobilised on a glass slide a dozen of micrometers away from the cell shows stable temperature readings (Ref. ND).

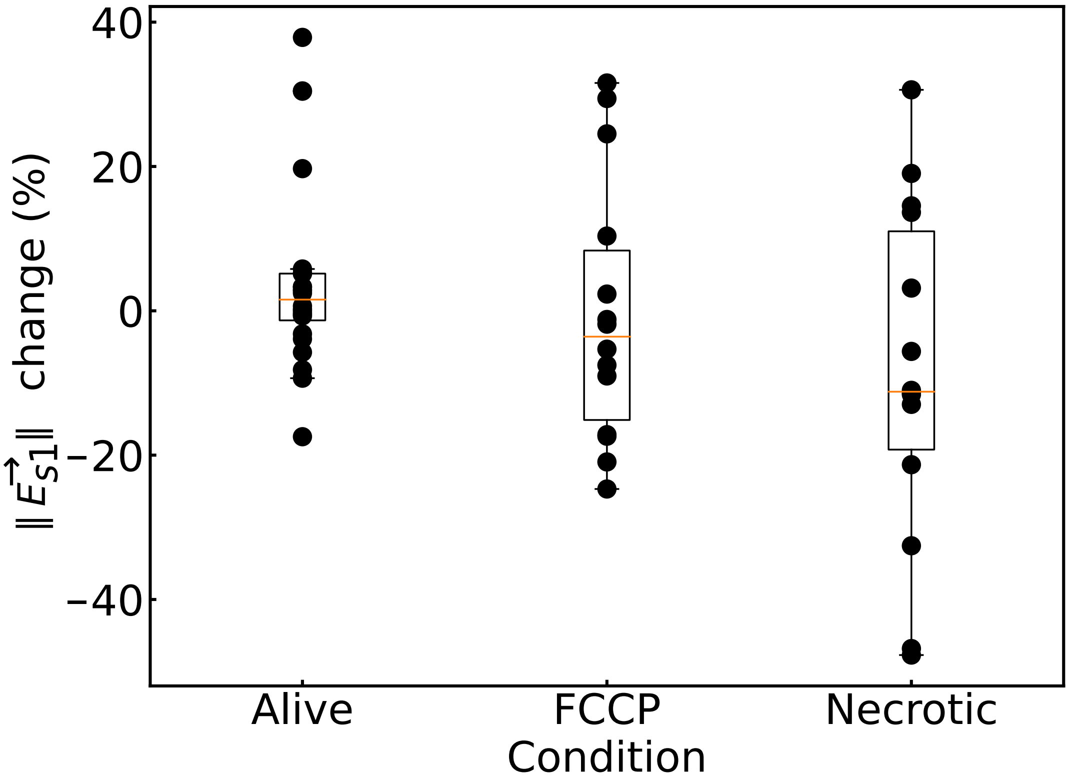

Figure S. 13: $\| \overrightarrow{E_{s1}} \|$ changes in unfunctionalised NDs inside macrophages (necrosis and FCCP). The percentage change is relative to the first measurement done inside the cell without FCCP treatment. Each point represent an intracellular measurement, the box is from the first to the third quartile with the orange line being the median. The whisker lengths are 1.5 times the inter-quartile range. N=6 cells for necrosis, N=4 for FCCP (5-9 measurements per cell). The necrotic condition includes treated and untreated cells (see figure 2 and 3 in the main text for details).

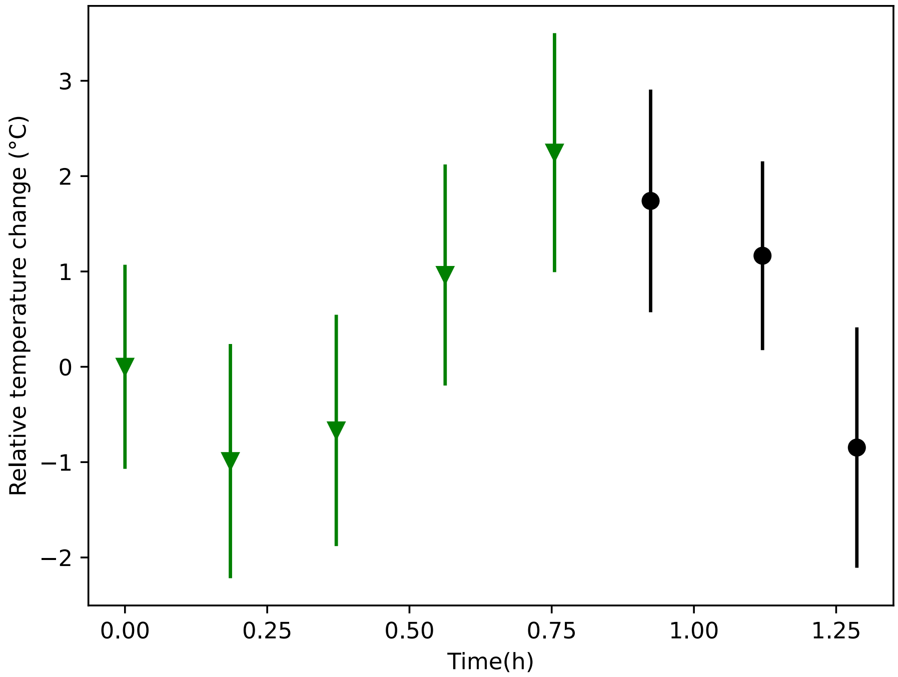

Figure S. 14: Intracellular temperature sensing using dipeptide functionalised NDs (CW ODMR). Green untreated, black FCCP.

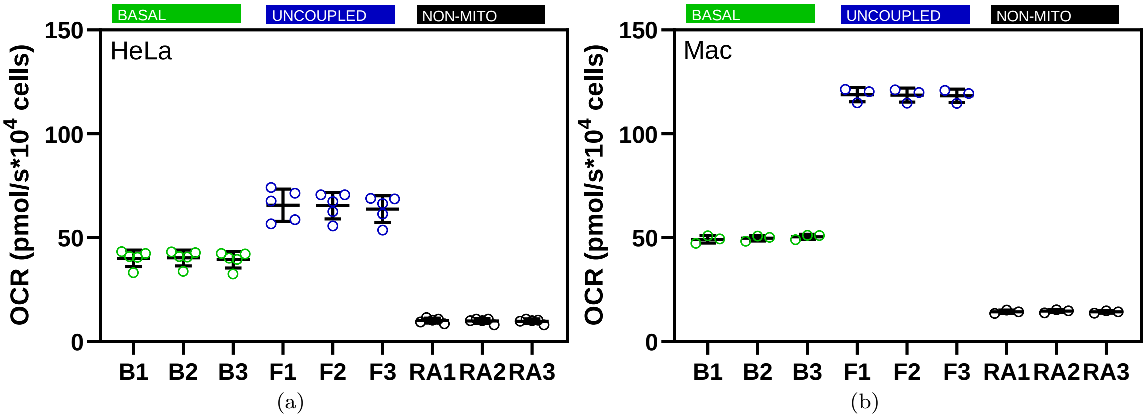

Figure S. 15: Comparative respirometry in HeLa cells (a) and primary human macrophages (b). Cells were plated into XFe96 microplates in either Dulbecco's Modified Eagle Medium (DMEM) (a) or RPMI (b) medium supplemented with 10mM glucose, 1mM pyruvate, and 2mM glutamine. Oxygen comsumption rates (OCR) were determined by plotting partial oxygen pressure against time using a XFe96 Flux Analyzer. Uncoupled respiration was determined by injecting carbonyl cyanide-p-trifluoromethoxyphenylhydrazone (FCCP, A:0.5µM, B:2.4µM). Non-mitochondrial respiration was determined by injecting both rotenone (complex I inhibitor, 0.5µM) and antimycin A (complex III inhibitor, 0.5µM). Data+/-SD from single experiments performed in five (a) or three (b) technical replicates are shown. B1-3: basal respiration, F1-3: respiration after FCCP, RA1-3, respiration after rotenone and antimycin A.

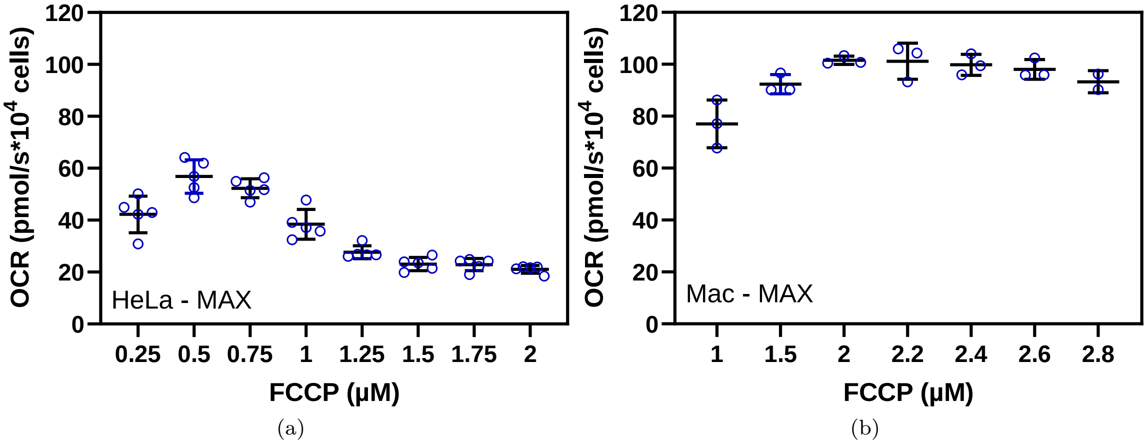

Figure S. 16: Mitochondrial uncoupling in response to FCCP in HeLa cells (a) and primary macrophages (b). Cells were plated into XFe96 microplates in either DMEM (a) or RPMI (b) medium supplemented with 10mM glucose, 1mM pyruvate, and 2mM glutamine. Oxygen comsumption rates (OCR) were determined by plotting partial oxygen pressure against time using a XFe96 Flux Analyzer. Uncoupled respiration was determined by injecting increasing concentrations of carbonyl cyanide-p-trifluoromethoxyphenylhydrazone (FCCP). Maximal respiration rates were calculated by subtracting non-mitochondrial respiration after injection of both rotenone (complex I inhibitor, 0.5µM) and antimycin A (complex III inhibitor, 0.5µM). Data+/-SD from single experiments performed in five (a) or three (b) technical replicates are shown.

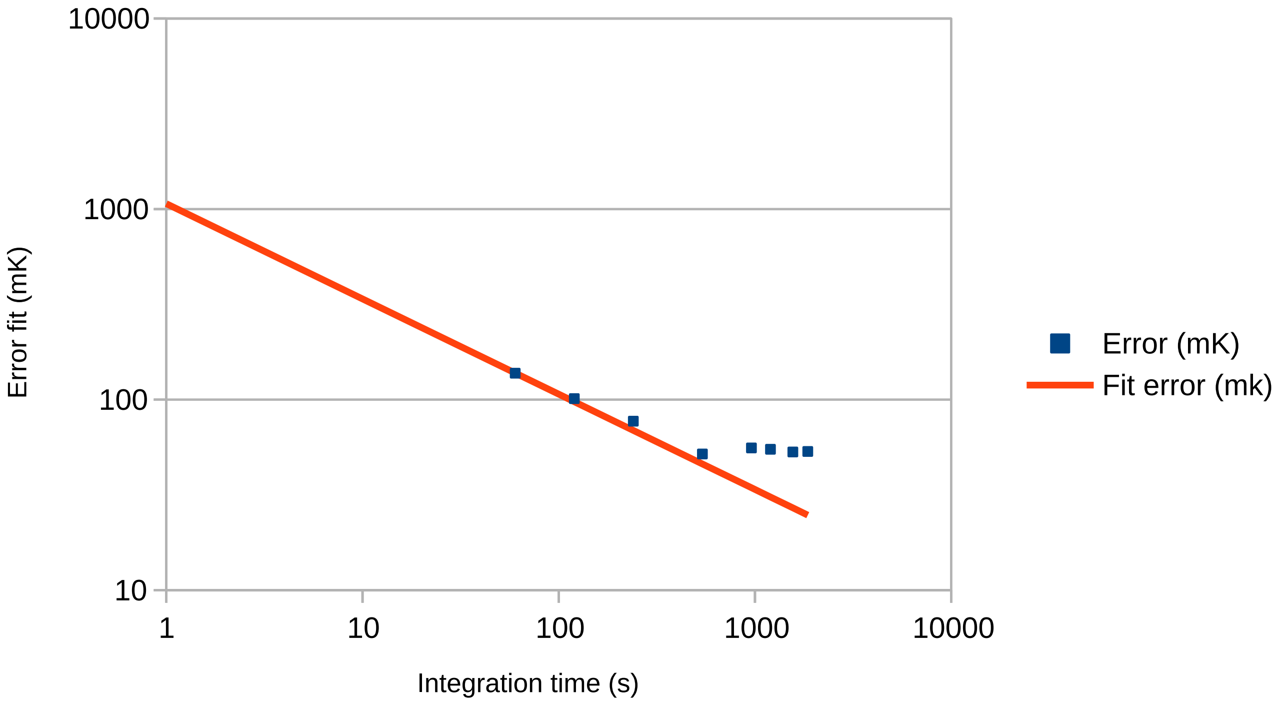

Figure S. 17: Uncertainty on the frequency of the sine fit on TE oscillations versus integration time. The fitting function is $y = 1068/\sqrt{time}$.

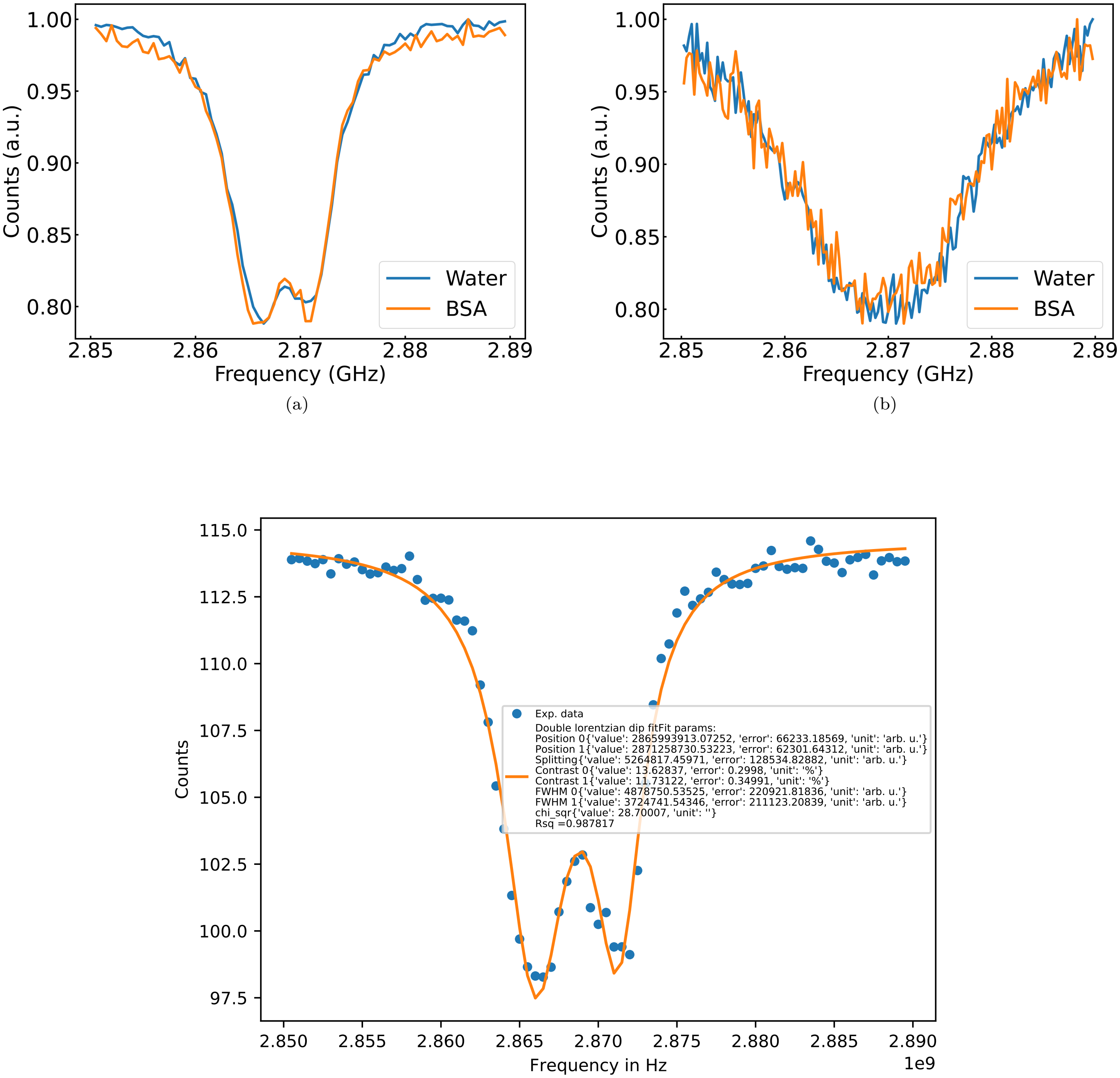

(a) (b)

(c)

Figure S. 18: ODMR spectra comparison of the same CVD NDs in water and in BSA (a: ND A; b: ND B). (c) Fitting of the ND A spectra.

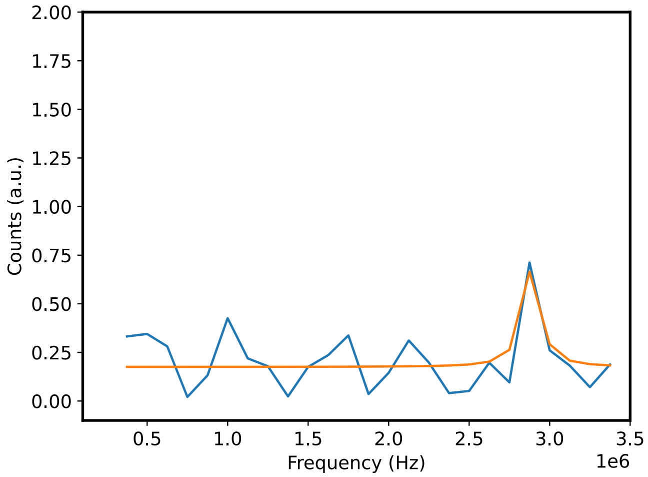

(a)

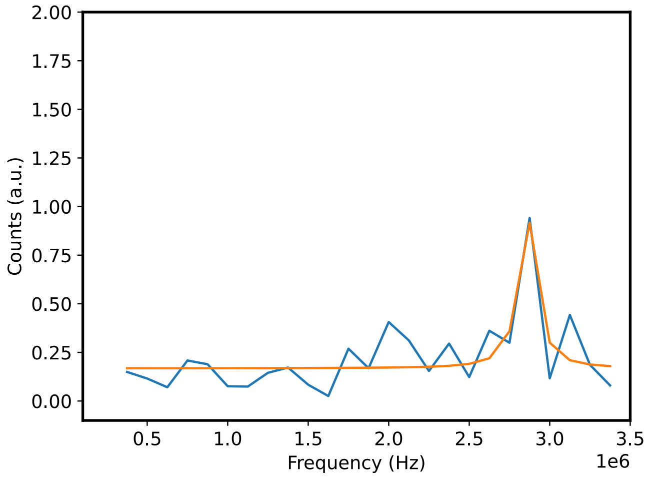

(b)

Figure S. 19: Comparison TE FFTs of a CVD NDs in water (left) and BSA (right). The peak centres from the fit are: 2886 KHz (± 66 KHz) and 2860 KHz (± 39 KHz)

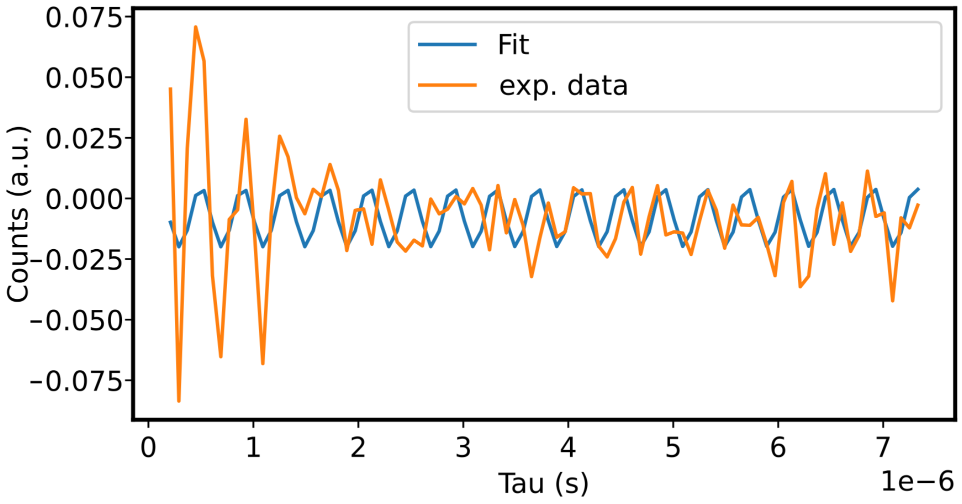

(a)

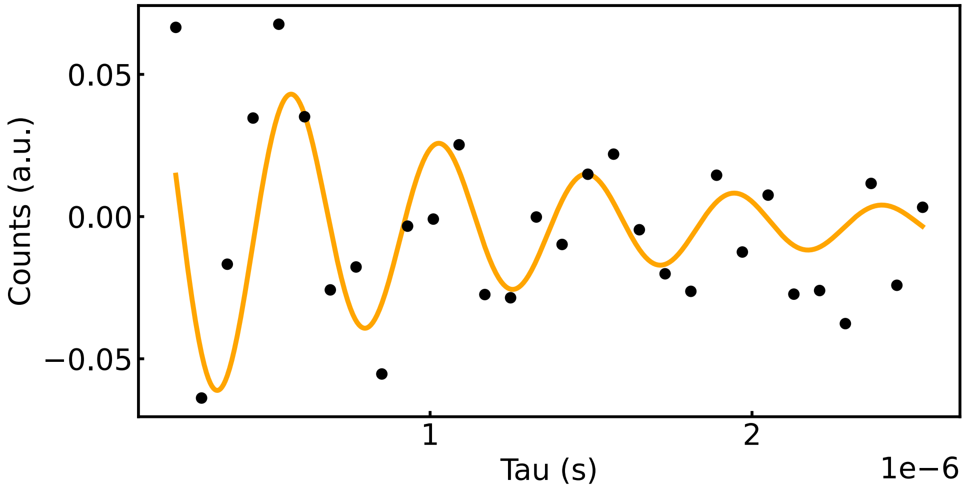

(b)

Figure S. 20: Example of RAP-TE measurements inside cells using CVD NDs (see figure 5 and experimental section in the main text for details).

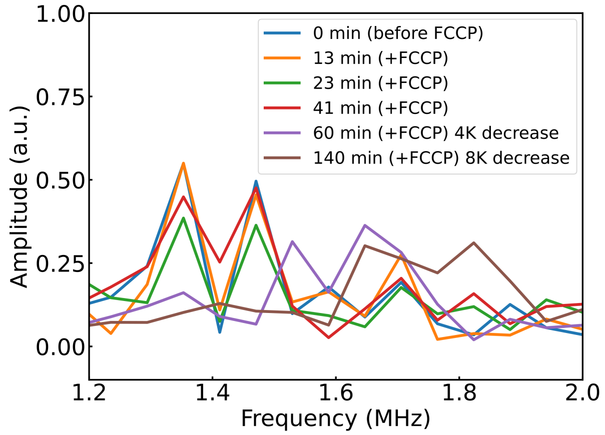

Figure S. 21: FFT of multiple RAP-TE measurements for a macrophage in different conditions, the two peaks correspond to two different NVs inside the CVD ND.

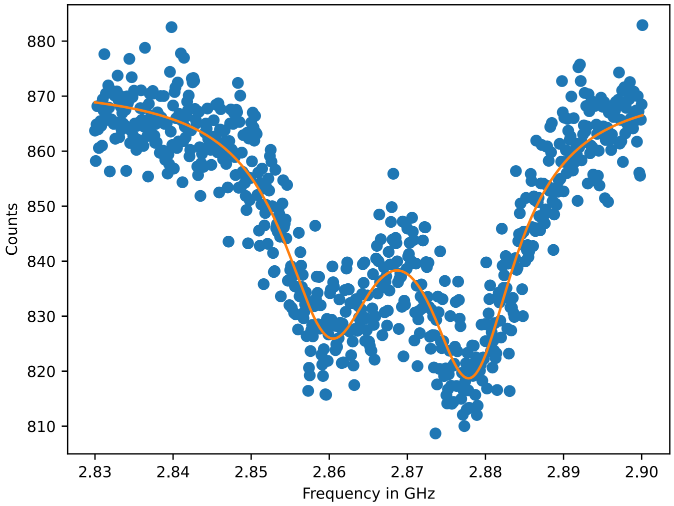

(a)

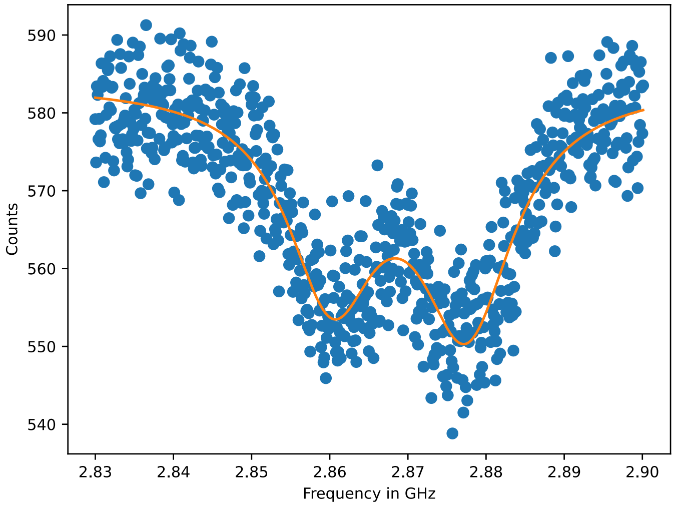

(b)

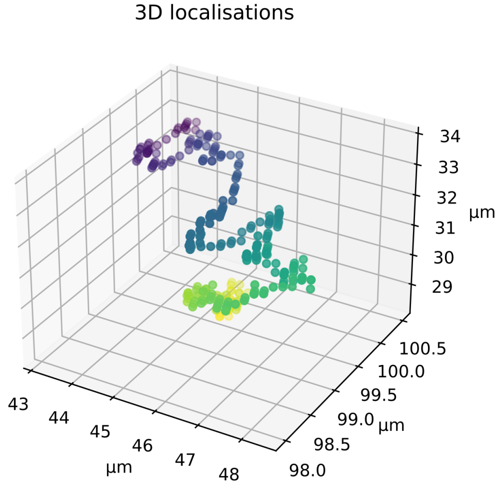

(c)

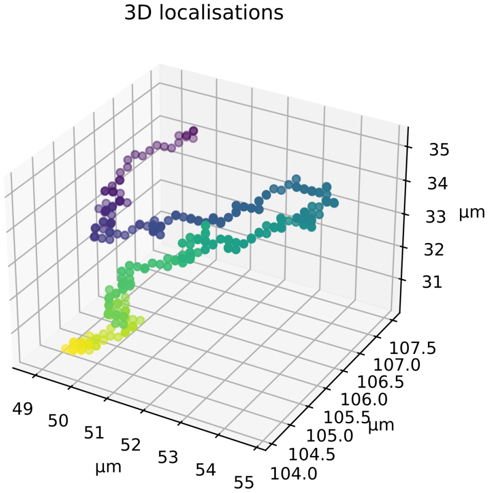

(d)

Figure S. 22: ODMR spectra comparison of the same ND in 98% glycerol at two different time points (+15 min difference between (a) and (b). Below: localization of the ND in glycerol during the measurements ((c) for (a) and (d) for (b)). The purple spot is the start. 2 min duration for each measurement

References

[1] Y. Mindarava, R. Blinder, C. Laube, W. Knolle, B. Abel, C. Jentgens, J. Isoya, J. Scheuer, J. Lang, I. Schwartz, B. Naydenov, F. Jelezko, *Carbon* **2020**, *170* 182.

[2] E. Mayerhoefer, H. Parajuli, M.-R. Cimpan, D. E. Costea, H. N. Dongre, A. Krueger, *Angewandte Chemie International Edition* **2025**, *n/a*, n/a e202501202.

[3] V. Merz, J. Lenhart, Y. Vonhausen, M. E. Ortiz-Soto, J. Seibel, A. Krueger, *Small* **2019**, *15*, 48 1901551.

[4] S. Bhagavantam, D. Narayana Rao, *Nature* **1948**, *161*, 4097 729.

[5] S. Whitehead, W. Hackett, *Proceedings of the Physical Society* **1939**, *51*, 1 173.

[6] Y.-H. Yu, R.-Z. Zhang, Y. Xu, X.-Q. Chen, H. Zheng, Q. Li, R.-B. Liu, X.-Y. Pan, D. Budker, G.-Q. Liu, *Phys. Rev. Appl.* **2024**, *21* 044051.

[7] F. Dolde, H. Fedder, M. W. Doherty, T. Nöbauer, F. Rempp, G. Balasubramanian, T. Wolf, F. Reinhard, L. C. Hollenberg, F. Jelezko, et al., *Nature Physics* **2011**, *7*, 6 459.

[8] T. Mittiga, S. Hsieh, C. Zu, B. Kobrin, F. Machado, P. Bhattacharyya, N. Z. Rui, A. Jarmola, S. Choi, D. Budker, N. Y. Yao, *Phys. Rev. Lett.* **2018**, *121* 246402.

[9] E. Van Oort, M. Glasbeek, *Chemical Physics Letters* **1990**, *168*, 6 529.

[10] H. T. Dinani, E. Munoz, J. R. Maze, *Nanomaterials* **2021**, *11*, 2.

[11] V. Petrakova, A. Taylor, I. Kratochvilova, F. Fendrych, J. Vacik, J. Kucka, J. Stursa, P. Cigler, M. Ledvina, A. Fiserova, et al., *Advanced Functional Materials* **2012**, *22*, 4 812.

[12] M. V. Hauf, B. Grotz, B. Naydenov, M. Dankerl, S. Pezzagna, J. Meijer, F. Jelezko, J. Wrachtrup, M. Stutzmann, F. Reinhard, J. A. Garrido, *Phys. Rev. B* **2011**, *83* 081304.

[13] S. Stehlik, T. Petit, H. A. Girard, A. Kromka, J.-C. Arnault, B. Rezek, *Journal of nanoparticle research* **2014**, *16* 1.

[14] S. Mahanta, P. Vallejo-Ramirez, N. Karedla, P. Puczkarski, M. Krishnan, *Proceedings of the National Academy of Sciences of the United States of America* **2022**, *119*, 49 e2209955119.

[15] S. H. Behrens, D. G. Grier, *The Journal of Chemical Physics* **2001**, *115*, 14 6716.

[16] M. Fujiwara, A. Dohms, K. Suto, Y. Nishimura, K. Oshimi, Y. Teki, K. Cai, O. Benson, Y. Shikano, *Phys. Rev. Res.* **2020**, *2* 043415.

[17] V. M. Acosta, E. Bauch, M. P. Ledbetter, A. Waxman, L.-S. Bouchard, D. Budker, *Physical review letters* **2010**, *104*, 7 070801.

[18] P. J. Vetter, A. Marshall, G. T. Genov, T. F. Weiss, N. Striegler, E. F. Großmann, S. Oviedo-Casado, J. Cerrillo, J. Prior, P. Neumann, F. Jelezko, *Phys. Rev. Applied* **2022**, *17* 044028.
